# A Novel Azaphilone Muyophilone A From the Endophytic Fungus *Muyocopron laterale* 0307-2

**DOI:** 10.3389/fchem.2021.734822

**Published:** 2021-08-24

**Authors:** Chao Yuan, Yuhua Guo, Ke Wang, Zhunian Wang, Longfei Li, Huajie Zhu, Gang Li

**Affiliations:** ^1^ Tropical Crops Genetic Resources Institute, Chinese Academy of Tropical Agricultural Sciences CATAS, Haikou, China; ^2^ Haikou Experimental Station, Chinese Academy of Tropical Agricultural Sciences CATAS, Haikou, China; ^3^ Department of Natural Medicinal Chemistry and Pharmacognosy, School of Pharmacy, Qingdao University, Qingdao, China; ^4^ College of Pharmacy, Hebei University, Baoding, China

**Keywords:** muyocopron, endophytes, azaphilones, ECD, VCD

## Abstract

Two known azaphilone derivatives, 4,6-dimethylcurvulinic acid (**1**) and austdiol (**2**), and their novel heterotrimer, muyophilone A (**3**), were isolated and identified from an endophytic fungus, *Muyocopron laterale* 0307-2. Their structures and stereochemistry were established by extensive spectroscopic analyses including HRMS, NMR spectroscopy, electronic circular dichroism (ECD) and vibrational circular dichroism (VCD) spectroscopic methods, as well as single crystal X-ray diffraction. In the structure of **3**, two compound **2**-derived azaphilone units were connected through an unprecedented five-membered carbon bridge which was proposed to be originated from compound **1**. Compound **3** represents the first example of azaphilone heterotrimers.

## Introduction

Azaphilones or azaphilonoids, a large family of naturally occurring fungal polyketides, have attracted considerable attention owing to their diverse structures and intriguing biological activities (Pavesi et al., 2021). Since the discovery of the best known fungal mycotoxin citrinin in 1931 (Hetherington and Raistrick, 1931), more than 600 azaphilones have been isolated and identified from diverse fungal genera, such as *Penicillium*, *Talaromyces*, *Aspergillus*, and *Chaetomium* species (Osmanova et al., 2010; Gao et al., 2013; Chen et al., 2020). Their structures are typically characterized by the presence of a pyrone-quinone bicyclic skeleton and a quaternary carbon center (Osmanova et al., 2010; Gao et al., 2013; Chen et al., 2020). The substitution and cyclization of different side chains, as well as the polyketide dimerization, greatly contribute to the structural diversity and complexity of azaphilones (Yin et al., 2017). Further incorporation of amines by the exchange of pyrane oxygen for nitrogen affords red or purple vinylogous*γ*-pyridones and also increases the number of azaphilones (Akihisa et al., 2005; Wei and Yao, 2005). Azaphilones exhibited a large range of biological activities, such as antimicrobial, cytotoxic, antioxidant, antiviral, and anti-inflammatory activities (Osmanova et al., 2010; Gao et al., 2013; Chen et al., 2020).

During our continuing search for biologically active secondary metabolites from fungal endophytes harbored in the medicinal plant *Blumea balsamifera* (Yuan et al., 2019), an endophyte *Muyocopron laterale* 0307-2 was isolated and chemically investigated. Three azaphilones including two known ones, 4,6-dimethylcurvulinic acid (**1**) and austdiol (**2**), and their novel trimeric derivative, muyophilone A (**3**), were obtained. By carefully searching azaphilone structures and to the best of our knowledge (Osmanova et al., 2010; Gao et al., 2013; Chen et al., 2020), the presence of a polysubstituted five-membered 1,3-diketone in compound **3** is unprecedented among azaphilones and their dimers or trimers (Figure S1). Here, we report their isolation, structural elucidation, as well as proposed biosynthetic pathway.

## Results and Discussion

**FIGURE 1:**
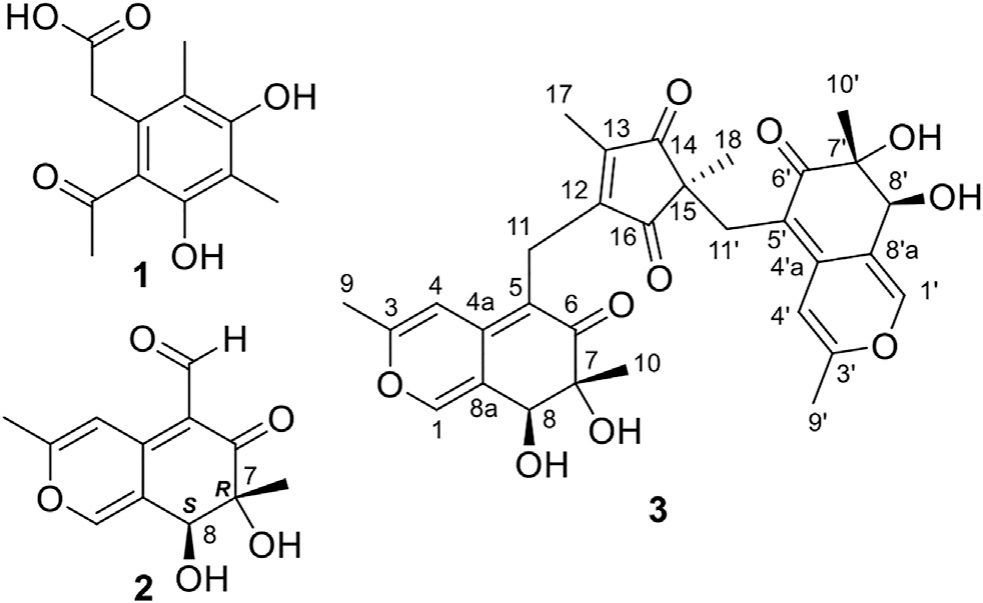
The structures of compounds **1**-**3**.

Compound **1** (Figure 1) was obtained as a white powder and compound **2** (Figure 1) was isolated as a yellow powder. They were identified as known azaphilones, 4,6-dimethylcurvulinic acid and austdiol, respectively, based on the comparison of their ^1^H and ^13^C NMR data with those reported in the literature (Supplementary Figures S2, S3) (Liu et al., 2017; de Oliveira et al., 2019). The absolute configuration of **2** was further confirmed to be 7*R*, 8*S* by single crystal X-ray diffraction and ECD calculation (Supplementary Figures S4, S5) (Presti et al., 2003).

Compound **3** (Figure 1) was also obtained as yellow powder. Its molecular formula was established as C_31_H_32_O_10_ by analysis of ESI-HRMS at *m/z* 565.2066 [M + H]^+^ (Supplementary Figure S12). The ^1^H NMR spectrum (Supplementary Figure S6) showed the presence of six singlet methyls (*δ*_H_ 0.99, 1.06, 1.09, 1.93, 2.21, and 2.23), two methylenes (*δ*_H_ 2.49, 1H, d, *J* = 14.0 Hz; *δ*_H_ 2.67, 1H, d, *J* = 14.0 Hz; *δ*_H_ 3.32, 1H, d, *J* = 15.5 Hz; *δ*_H_ 3.41, 1H, d, *J* = 15.5 Hz), two oxygenated methines (*δ*_H_ 4.33, and 4.52), and four olefinic or aromatic protons (*δ*_H_ 6.28, 6.50, 7.39, and 7.44). The ^1^H and ^13^C NMR data (Table 1 and Supplementary Figure S7) in combination with HSQC spectrum (Supplementary Figure S8) confirmed the presence of six methyls, two methylenes, two oxygenated methines, three quaternary carbons (two oxygenated), and 14 olefinic/aromatic carbons (four oxygenated), together with four ketones. These data accounted for all ^1^H and ^13^C NMR resonances.

**TABLE 1:** ^1^H (500 MHz) and ^13^C (125 MHz) NMR Data of Compound **3** in CD_3_OD (*δ* in ppm).

No	*δ*_C_	*δ*_H_ (*J* in Hz)	no	*δ*_C_	*δ*_H_ (*J* in Hz)
1	146.1	7.44 (1H, s)	1′	146.1	7.39 (1H, s)
3	161.7		3′	161.5	
4	105.7	6.50 (1H, s)	4′	105.8	6.28 (1H, s)
4a	145.1		4′a	145.5	
5	110.6		5′	110.4	
6	199.2		6′	199.5	
7	77.7		7′	77.4	
8	72.9	4.52 (1H, s)	8′	72.9	4.33 (1H, s)
8a	122.1		8′a	121.9	
9	19.9	2.23 (3H, s)	9′	19.8	2.21 (3H, s)
10	18.9	1.06 (3H, s)	10′	19.1	0.99 (3H, s)
11a	20.9	3.32 (1H, d, 15.5)	11′a	31.4	2.67 (1H, d, 14.0)
11b		3.41 (1H, d, 15.5)	11′b		2.49 (1H, d, 14.0)
12	156.9		16	207.2	
13	155.8		17	9.6	1.93 (3H, s)
14	207.8		18	18.6	1.09 (3H, s)
15	51.3				

**FIGURE 2:**
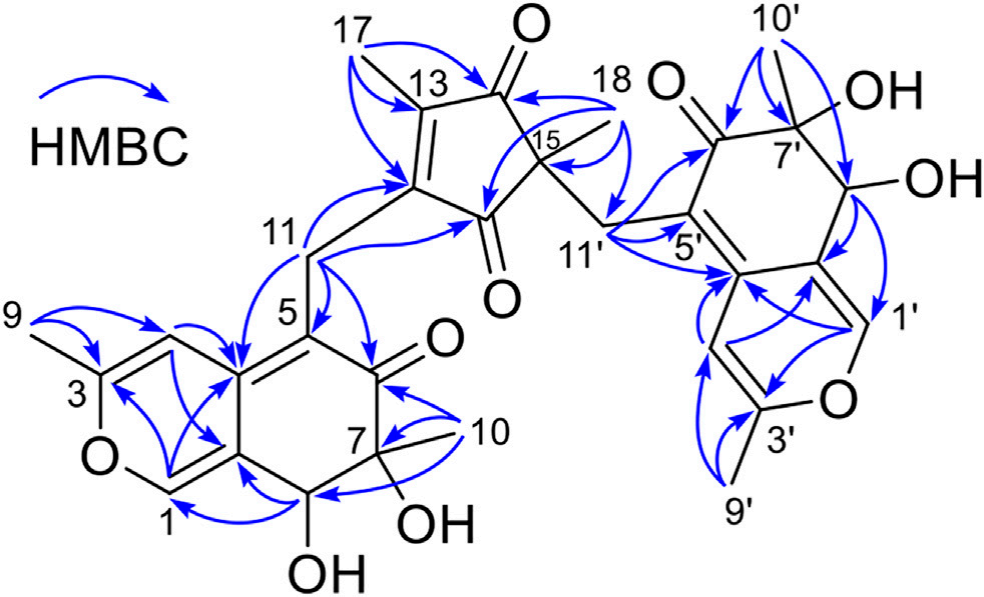
The key HMBC correlations of compound **3**.

The planar structure of **3** was constructed by detailed analysis of HMBC spectrum (Supplementary Figure S9). Key HMBC correlations from H_3_-9 to C-3 and C-4, from H-4 to C-4a and C-8a, and from H-1 to C-3 and C-4a (Figure 2), coupled with the requirement of chemical shifts of C-1 (*δ*_C_ 146.1) and C-3 (*δ*_C_ 161.7) identified a *γ*-pyran ring with a methyl at C-3. Further analysis of key HMBC correlations of H_3_-10/C-6, H_3_-10/C-7, H_3_-10/C-8, H-8/C-8a, H_2_-11/C-4a, H_2_-11/C-5, and H_2_-11/C-6 (Figure 2), as well as the chemical shifts of C-6 (*δ*_C_ 199.2), C-7 (*δ*_C_ 77.7), and C-8 (*δ*_C_ 72.9) demonstrated an azaphilonoid moiety. This substructure was similar to that of co-isolated austdiol (**2**), except for the C-11 methylene in **3** instead of aldehyde group in **2**.

A five-membered carbon ring, 1,3-diketone moiety, was further verified and connected to C-11 on the basis of the key HMBC correlations of H_2_-11 with C-12 and C-16, of H_3_-17 with C-12, C-13, and C-14, and of H_3_-18 with C-14, C-15, and C-16 (Figure 2), as well as the chemical shifts of C-14 (*δ*_C_ 207.8) and C-16 (*δ*_C_ 207.2) (Table 1). Another austdiol (**2**)-derived azaphilone moiety was confirmed to be present in the structure of **3** by HMBC correlations as shown in Figure 2. It was linked to C-15 of the five-membered ring by the key HMBC correlations of H_3_-18 with C-11′, which was also consistent with the MS requirement. The above results indicated that compound **3** is anazaphilone that contained two austdiol (**2**)-derived units.

Considering the same biosynthetic origin and the classical structural characteristics of azaphilone dimers or trimers (Supplementary Figure S1), the stereochemistry of azaphilone monomers in **3** should be same to that of 7*R*,8*S*-austdiol (**2**), revealing a 7*R*,8*S,*7′*R*,8′*S* configuration for **3**. In accordance of our previous computational study on absolute configurations assignments for natural products (Cao et al., 2019; Ren et al., 2017; Xu et al., 2016). the electronic circular dichroism (ECD) calculations and vibrational circular dichroism (VCD) (Mazzeo et al., 2013; Mándi and Kurtán, 2019; Mazzeo et al., 2017; Ding et al., 2020) were performed to clarify the absolute configuration of C-15 of **3**.

**Scheme 1:**
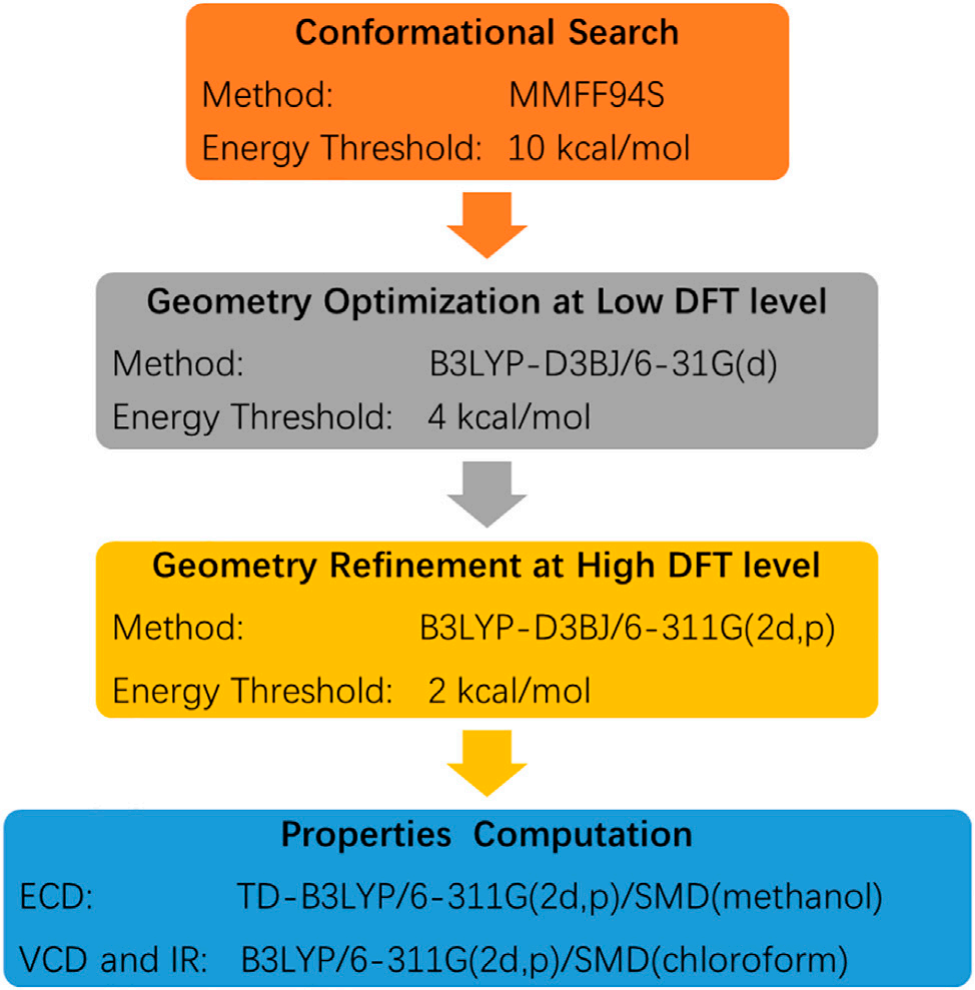
The procedure for the ECD and VCD computations.

**FIGURE 3:**
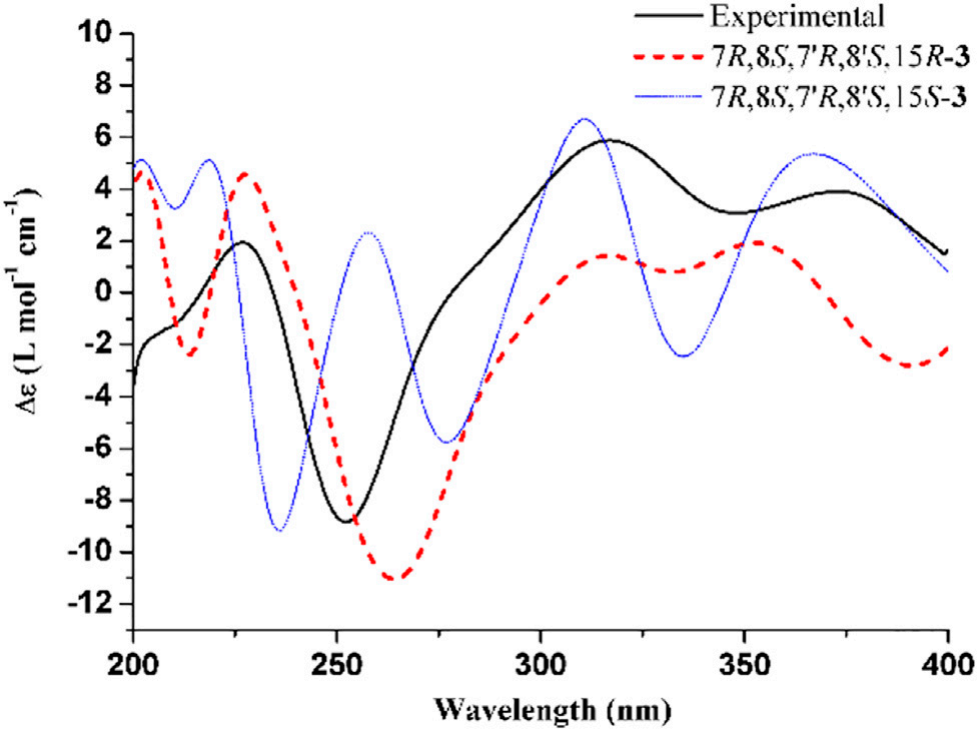
Experimental ECD spectrum of **3** and calculated ECD spectra for (7*R*,8*S*,7′*R*,8′*S*,15*R*)-**3** and (7*R*,8*S*,7′*R*,8′*S*,15*S*)-**3.** The band width was set to 0.18 eV.

**FIGURE 4:**
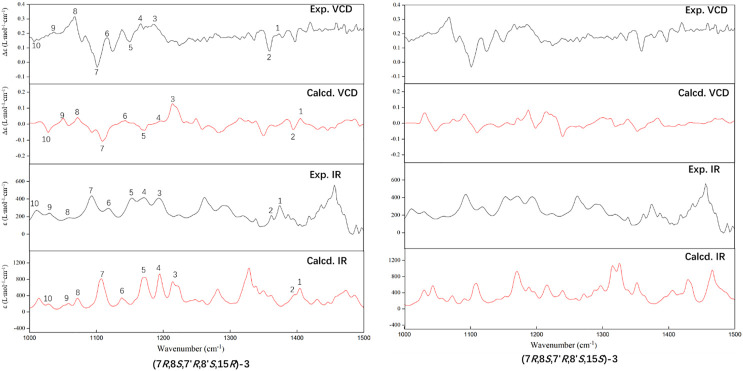
Comparison of the calculated (Calcd.) VCD and IR spectra of (7*R*,8*S*,7′*R*,8′*S*,15*R*)-**3** and (7*R*,8*S*,7′*R*,8′*S*,15*S*)-**3** with the experimental (Exp.) spectra of **3**. The band half-width is 4 cm^−1^.

The experimental ECD and VCD conditions are shown in the experimental section in the Supplementary Material. The procedure for the ECD and VCD computation is shown in Scheme 1. Conformational searches for compound **3** were first performed using the MMFF94S force field (Halgren, 1999) and the resulted conformers within 0–10 kcal/mol (Supplementary Table S1) were optimized through density functional theory (DFT). The benchmark performed suggests the dispersion-corrected functional B3LYP-D3BJ owns a high accuracy, and the method is proposed for biochemically relevant systems (Pracht and Grimme, 2021; Katsyuba et al., 2019). Those conformers within 0–10 kcal/mol were optimized through the B3LYP-D3BJ/6-31G(d) level, and the optimized structures with relative energies ranging from 0 to 4 kcal/mol were further re-optimized at the B3LYP-D3BJ/6-311G (2d,p) level (Rassolov et al., 1998). The ECD computations were performed at the TDDFT/B3LYP/6-311G (2d,p)/SMD (methanol) level, and the VCD and IR computations were performed at the B3LYP/6-311G (2d,p)/SMD (chloroform) level (Marenich et al., 2009). All the computations are performed in the Gaussian09 programs (Frisch et al., 2009). The Cartesian coordinates of all conformers and corresponding energies are presented in the Supplementary Material. As showed in Figure 3, the calculated ECD spectrum of (7*R*,8*S*,7′*R*,8′*S*,15*S*)-**3** had a positive Cotton effect near 262 nm. However, the experimental ECD had a negative Cotton effect at 254 nm. In contrast, the (7*R*,8*S*,7′*R*,8′*S*,15*R*)-**3** had a negative Cotton effect at 265 nm. Therefore, ECD curve of (7*R*,8*S*,7′*R*,8′*S*,15*R*)-**3** is in good agreement with the experimental curve. The VCD study gives the consistent result, and the predicted vibrational modes 1 to 10 labelled in the Figure 4 for (7*R*,8*S*,7′*R*,8′*S*,15*R*)-**3** are in good agreement with the experimental results. Besides, the similarity factor (Bruhn et al., 2013; Rodriguez-Garcia et al., 2019) issued to quantify the degree of matching of VCD curves with the SpecDis software (Bruhn et al., 2017), and the value of (7*R*,8*S*,7′*R*,8′*S*,15*R*)-**3** is 0.5838 which is significantly higher than that for (7*R*,8*S*,7′*R*,8′*S*,15*S*)-**3** (0.4096). In conclusion, the absolute configuration of **3** is finally assigned as 7*R*,8*S*,7′*R*,8′*S*,15*R*.

**Scheme 2:**
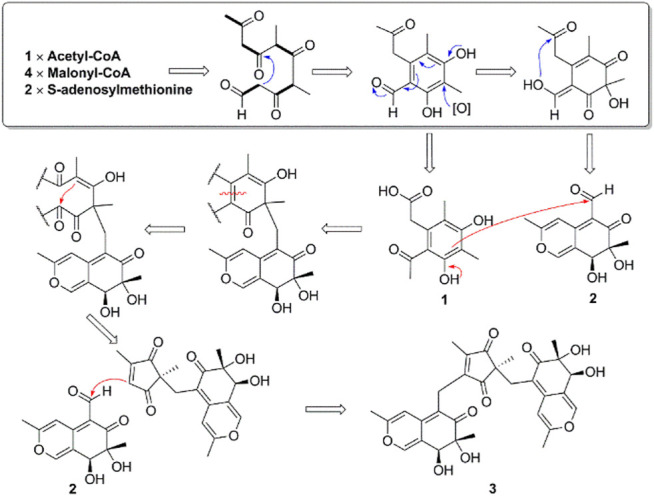
The Proposed Biosynthetic Pathway of Compounds **1–3**.

Compound **3** features a polysubstituted five-membered ring, 1,3-diketone, which acts as an unprecedented bridge with the attachment of two austdiol (**2**)-derived azaphilone wings. This structural feature is unprecedented among azaphilones and their dimers or trimers (Supplementary Figure S1). Based on the structural characteristics of **3** (Pavesi et al., 2021; Powers et al., 2019), its biosynthetic pathway is proposed as shown in Scheme 2. A linear PKS biosynthetic precursor was first constructed from a polyketide chain (pentaketide) followed by cyclization and oxidation to afford compound **1** or **2**. Further heterotrimerization between one molecule **1** and two molecules **2** formed the final product **3**. During this heterotrimerization, perhaps the most intriguing step is the proposed oxidation-rearrangement in the six-membered ring of **1** to produce the five-membered 1,3-diketone in **3**. Following the established bioassay methods in our laboratory, the novel compound muyophilone A (**3**) was evaluated for antibacterial activities (against *Staphylococcus aureus*, *Bacillus subtilis, Escherichia coli*, and *Pseudomonas aeruginosa*), and antifungal activity (against *Candida albicans*). Unfortunately, no inhibitory activities were observed.

## Conclusion

In summary, muyophilone A (**3**) represents a new family of azaphilone trimers featuring an unprecedented five-membered carbon bridge, expanding the structural diversity of azaphilones. The unique biosynthetic pathway of **3** is worth unveiling in future study.

## Data Availability

The original contributions presented in the study are included in the article/Supplementary Material, further inquiries can be directed to the corresponding authors.
