# Supplementary material for: A Novel Azaphilone Muyophilone A From the Endophytic Fungus Muyocopron laterale 0307-2
Source: Front Chem. 2021 Aug 24;9:734822. doi: 10.3389/fchem.2021.734822 (PMC8417894; doi:10.3389/fchem.2021.734822)
Supplement: Supplementary file 1 [file Table1.DOCX]

**SUPPLEMENTARY MATERIAL**

**A Novel Azaphilone Muyophilone A from the Endophytic Fungus *Muyocopron laterale* 0307-2**

Chao Yuan*^§,1^*, Yuhua Guo*^§,2^*, Ke Wang*^3^*, Zhunian Wang*^1^*, Longfei Li*^4^*^*^, Huajie Zhu*^4^*^*^, and Gang Li*^3^*^*^

*^1^Tropical Crops Genetic Resources Institute, Chinese Academy of Tropical Agricultural Sciences CATAS, Haikou 571101, People's Republic of China, ^2^Haikou Experimental Station, Chinese Academy of Tropical Agricultural Sciences CATAS, Haikou 571101, People's Republic of China, ^3^Department of Natural Medicinal Chemistry and Pharmacognosy, School of Pharmacy, Qingdao University, Qingdao 266071, People’s Republic of China, ^4^College of Pharmacy, Hebei University, Baoding 071002, Hebei, People’s Republic of China*

^§^CY and YHG contributed equally to this work.

Correspondence authors:

lilongfei@hbu.edu.cn; zhuhuajie@hotmail.com; gang.li@qdu.edu.cn

| **Table of Contents** | **Pages** |
| --- | --- |
| **Experimental section** | **3** |
| **General Experimental Procedures.** | **3** |
| **Fungal Material.** | **3** |
| **Extraction and Isolation.** | **4** |
| **X-ray Diffraction Crystallographic Analysis of Austdiol (2).** | **5** |
| **Biological Assay.** | **5** |
| **Figure S1.** Representative azaphilone dimers or trimers from fungi. | **6** |
| **Figure S2.** ^1^H (500 MHz) and ^13^C (125 MHz) NMR spectra of compound **1** (CD_3_OD). | **7** |
| **Figure S3.** ^1^H (500 MHz) and ^13^C (125 MHz) NMR spectra of compound **2** (DMSO-*d*_6_). | **8** |
| **Figure S4.** Experimental and calculated ECD spectra of compound **2**. | **9** |
| **Figure S5.** X-ray diffraction of compound **2**. | **10** |
| **Figure S6.** ^1^H NMR spectrum of compound **3** (500 MHz, CD_3_OD). | **11** |
| **Figure S7.** ^13^C NMR spectrum of compound **3** (125 MHz, CD_3_OD). | **12** |
| **Figure S8.** HSQC spectrum of compound **3** (500 MHz, CD_3_OD). | **13** |
| **Figure S9.** HMBC spectrum of compound **3** (500 MHz, CD_3_OD). | **14** |
| **Figure S10.** ROESY spectrum of compound **3** (500 MHz, CD_3_OD). | **15** |
| **Figure S11.** IR spectrum of compound **3**. | **16** |
| **Figure S12.** Positive ESI-HRMS spectrum of compound **3**. | **17** |
| **Figure S13.** The individual ECD spectra of each conformation for (7*R*,8*S*,7'*R*,8'*S*,15*R*)-**3** at the B3LYP/6-311G(2d,p)/SMD (methanol) level. | **18** |
| **Figure S14.** The individual ECD spectra of each conformation for (7*R*,8*S*,7'*R*,8'*S*,15*S*)-**3** at the B3LYP/6-311G(2d,p)/SMD (methanol) level. | **19** |
| **Figure S15.** Fungal ITS sequence and phylogenetic tree. | **20** |
| **Figure S16.** Total conformer population of (7*R*,8*S*,7'*R*,8'*S*,15*R*)-**3**. | **21** |
| **Figure S17.** Total conformer population of (7*R*,8*S*,7'*R*,8'*S*,15*S*)-**3**. | **22** |
| **Table S1.** The calculated relative energies (△*E,* kcal/mol) at the MMFF94S level. The energy threshold is 10 kcal/mol. | **23** |
| **Table S2.** The Cartesian coordinates of conformers for (7*R*,8*S*,7'*R*,8'*S*,15*R*)-**3** and (7*R*,8*S*,7'*R*,8'*S*,15*S*)-**3** at the B3LYP-D3BJ/6-311G(2d,p) level. | **25** |
| **References** | **43** |

**Experimental section**

**General Experimental Procedures.**

Optical rotations were obtained using a JASCOP-2000 polarimeter. ESI-HRMS spectra were collected on an Agilent 6520 Q-TOF mass spectrometer. ECD spectra were recorded on an Applied Photophysics Chiral scan spectropolarimeter (experimental condition: solvent: methanol; pathlength: 1 mm).

IR and VCD spectra were measured on a BioTools ChiralIR-2X spectrophotometer (experimental condition: solvent: CDCl_3_; concentration: 48 mg/mL; anharmonicity factor: 1; photo elastic modulator (PEM): 0.25; spectral resolution: 4 cm^-1^; windows: Baf2; length of the spacer: 0.1 mm; measurement time: 12 hours).

NMR spectra were measured on a Bruker 500 MHz spectrometer. Silica gel (Qingdao Marine Chemical Factory, Qingdao, China) was used for column chromatography (CC).

The semi-preparative high performance liquid chromatography (HPLC) was performed on an Agilent 1260 system equipped with an RP-18 column (250 × 10 mm, YMC Park, 5 µm).

Thin layer chromatography (TLC) experiments were conducted with silica gel GF-254 pre-coated on glass plates (Qingdao Marine Chemical Factory, Qingdao, China). TLC spots were visualized under UV light at 254 nm and by spraying with 10% H_2_SO_4_ in alcohol followed by heating.

**Fungal Material.**

The fungal endophyte was isolated from the medicinal plant *Blumea balsamifera* collected from Danzhou, Hainan Province, People’s Republic of China. It was identified as *Muyocopron laterale* by internal transcribed spacer sequencing (Figure S14). The fungus was assigned the strain designation RO-190307-2, and was deposited at Tropical Crops Genetic Resources Institute, Chinese Academy of Tropical Agricultural Sciences CATAS, Hainan, People's Republic of China. For large-scale fermentation, the strain was cultured on potato dextrose agar for five days. Then, the fungal mycelia were inoculated in 30 flasks (500 mL) each containing 60 g rice and 80 mL distilled water. Finally, the cultures were incubated at 25 ºC for 40 days.

**Extraction and Isolation.**

The fermented material was extracted with the organic solvent ethyl acetate (EtOAc) for three times. All the organic solvent was collected and evaporated to give a crude extract (20.3 g). The extract was fractionated by CC on silica gel eluted with CH_2_Cl_2_-MeOH (100:0-0:100). The eluents were combined based on the analysis of TLC experiments, resulting in seven fractions (Fr.A-G).

Fr.B (6.2 g) from the eluent of CH_2_Cl_2_-MeOH (99:1) was separated by filtration and crystallized from MeOH to yield austdiol (**2**, purified 4.5 mg). The purity of compound **2** was confirmed by HPLC. Fr.C (3.3 g) also from CH_2_Cl_2_-MeOH (99:1) was subjected to sephadex LH-20 column chromatography eluting with MeOH to afford three subfractions (Fr.C1-C3). Purification of Fr.C3 by HPLC (MeOH-H_2_O, 63:37, 2.0 mL/min) yield 4,6-dimethylcurvulinic acid (**1**, *t*_R_=11.5min, 18.6 mg). Fr.D (8.0 g) from the eluent of CH_2_Cl_2_-MeOH (98:2) was subjected to Sephadex LH-20 column eluting with MeOH to provide two major subfractions (Fr.D1 and Fr.D2). Fr.D2 was further separated by Sephadex LH-20 (MeOH) to give one minor subfraction, which was finally purified by HPLC (MeOH-H_2_O, 72:28, 2.0 mL/min) to afford muyophilone A (**3**, *t*_R_ = 17.0 min, 19.2 mg).

*Austdiol (****2****):* yellow solid; [α]^20^_D_ +24.8718 (0.195 mg/mL, CHCl_3_), [α]^20^_546nm_ +203.5898 (0.195 mg/mL, CHCl_3_), [α]^20^_436nm_ +458.9744 (0.195 mg/mL, CHCl_3_), [α]^20^_365nm_ +145.1282 (0.195 mg/mL, CHCl_3_).

*Muyophilone A (****3****):* yellow solid; [α]^20^_D_ +39.4402 (0.863 mg/mL, CHCl_3_), [α]^20^_546nm_ +144.9615 (0.863 mg/mL, CHCl_3_), [α]^20^_436nm_ +333.8225 (0.863 mg/mL, CHCl_3_), [α]^20^_365nm_ +86.1197 (0.863 mg/mL, CHCl_3_); LC-UV *λ*_max_ (MeOH): 194, 236, 360 nm; IR *υ*_max_:3409.9, 2927.7, 2856.4, 1691.4, 1677.9, 1616.2, 1541.0; ECD (MeOH) *λ*: 227, 252, 316, 348, 373; ^1^H (500 MHz, CD_3_OD) and ^13^C (125 MHz, CD_3_OD) NMR data: see Table 1; ESI-HRMS *m/z*: 565.2066, [M+H]^+^ (calcd. for C_31_H_33_O_10_, *m/z* 565.2068, Δ -0.3539 ppm), *m/z* 587.1893, [M+Na]^+^ (calcd. for C_31_H_32_O_10_Na, *m/z* 587.1888, Δ 0.8515 ppm);

**X-ray Diffraction Crystallographic Analysis of Austdiol (2).**

The method was same to that previously reported.^1^ The Flack parameter was 0.03(15), and the absolute configuration was unambiguously assigned. Crystallographic data of austdiol (**2**) was deposited in the Cambridge Crystallographic Data Center (CCDC) with deposition numbers 2061708.

**Biological Assay.**

The antimicrobial activities of isolated compounds were evaluated by the disk diffusion method.^1^ Four bacteria *Staphylococcus aureus* (ATCC 6538), *Bacillus subtilis* (ATCC 9372)*, Escherichia coli* (ATCC 25922), and *Pseudomonas aeruginosa* (ATCC 27853), and one *Candida albicans* strain SC5314 were used in this study. Streptomycin and fluconazole were used as the positive controls for antibacterial test and antifungal evaluation, respectively.

**Figure S1.** Representative azaphilone dimers or trimers from fungi.

**Figure S2.**^1^H (500 MHz) and ^13^C (125 MHz) NMR spectra of compound **1** (CD_3_OD).


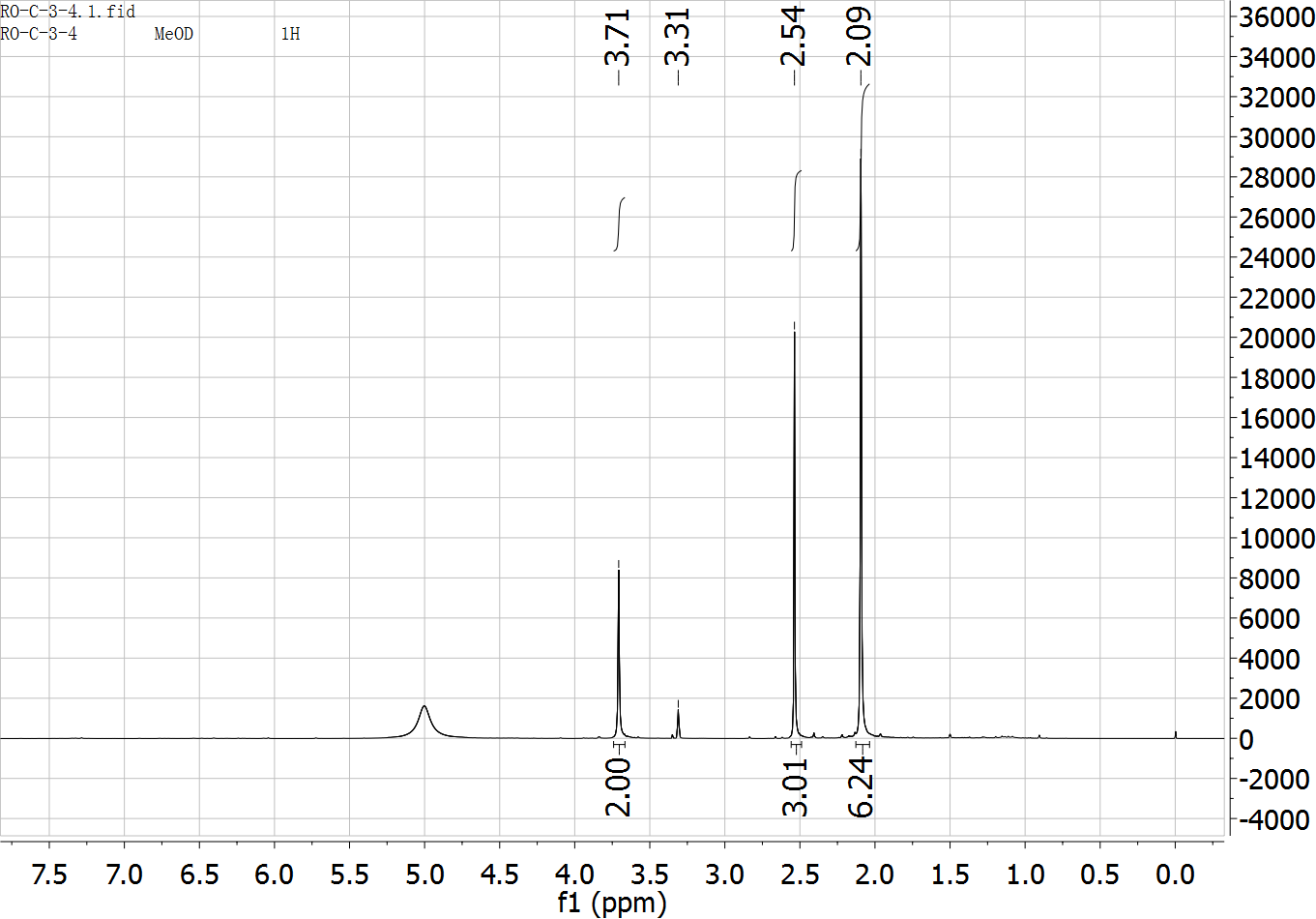


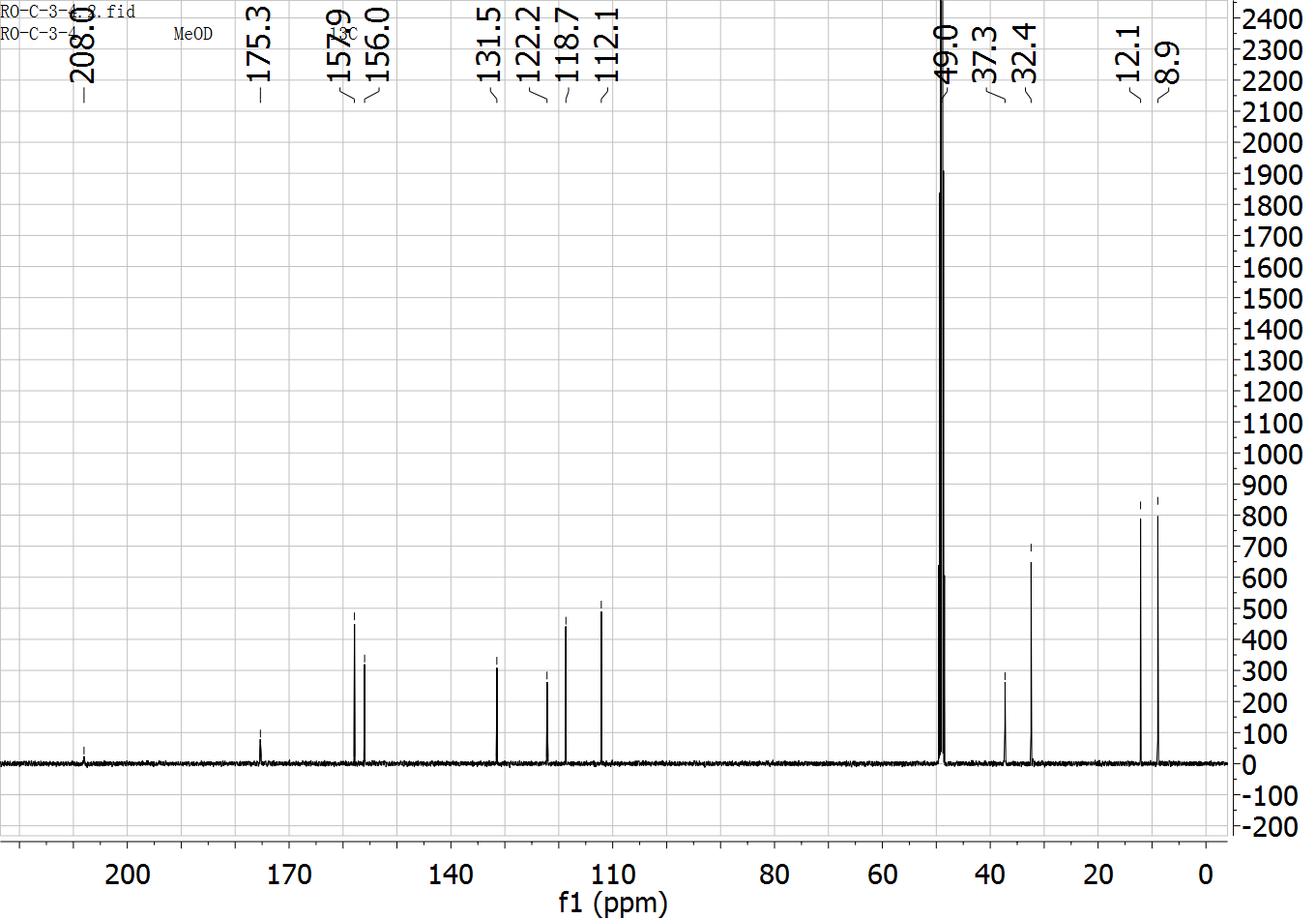


**Figure S3.**^1^H (500 MHz) and ^13^C (125 MHz) NMR spectra of compound **2** (DMSO-*d*_6_).


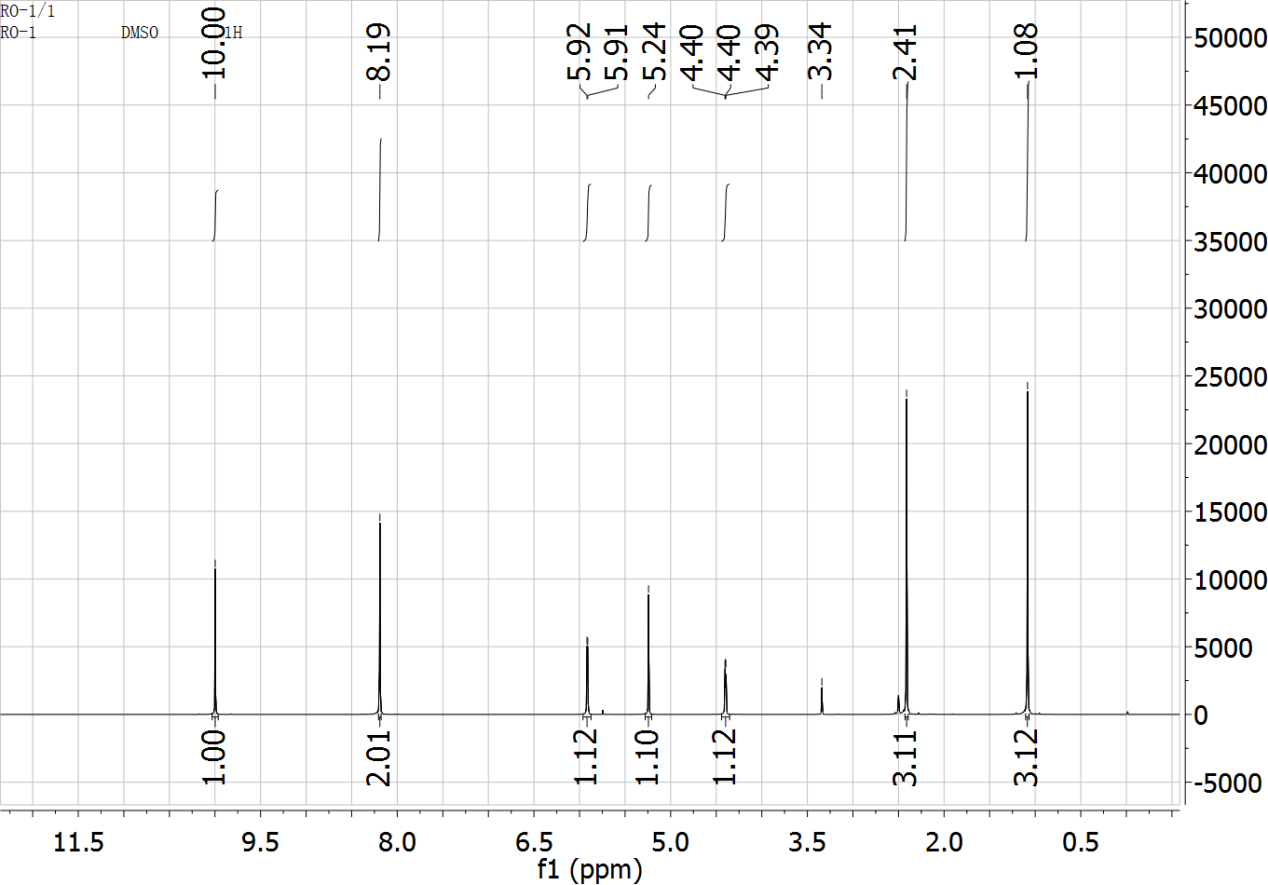


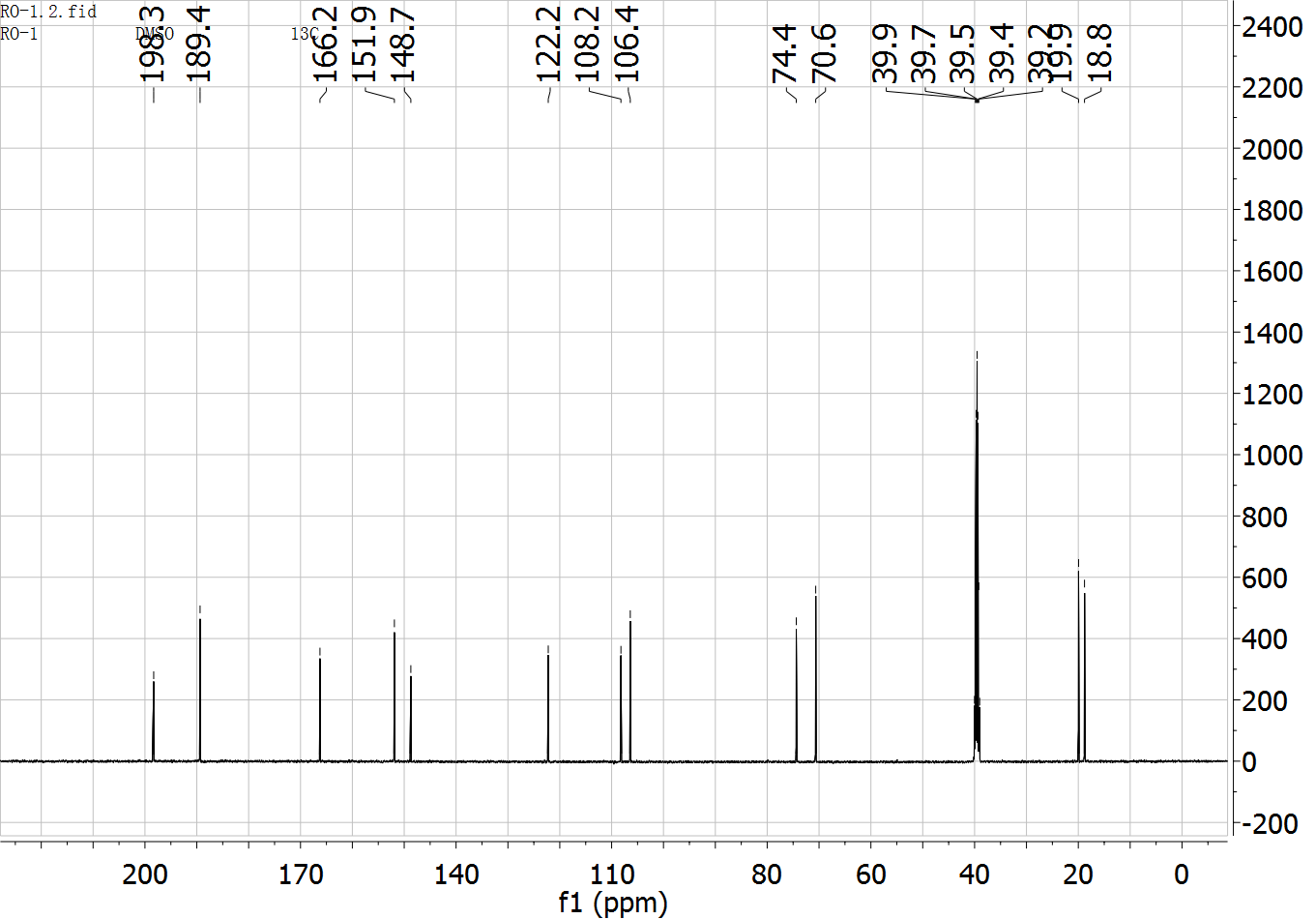


**Figure S4.** Experimental and calculated ECD spectra of compound **2**.

**Experimental ECD (0.4 mg/mL):**


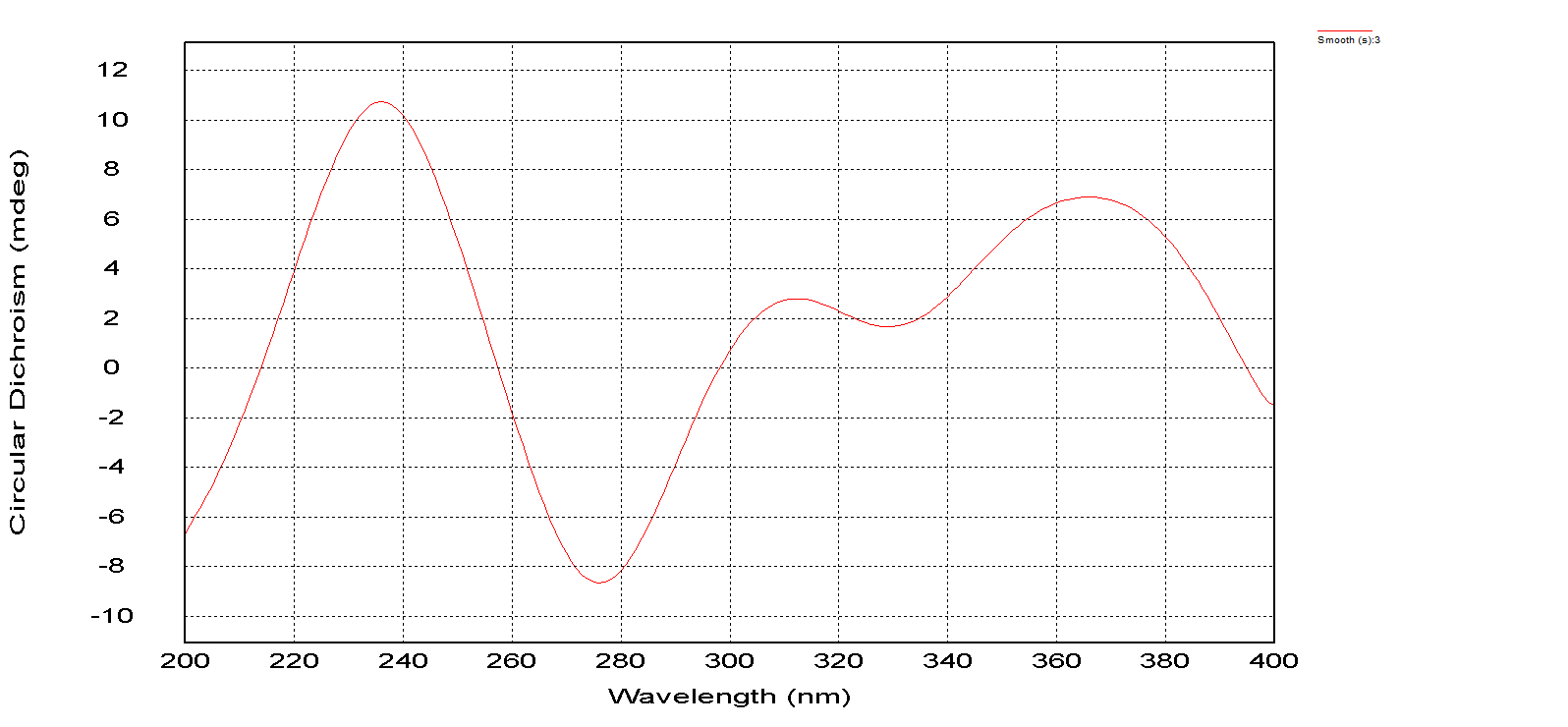


**Calculated ECD:**


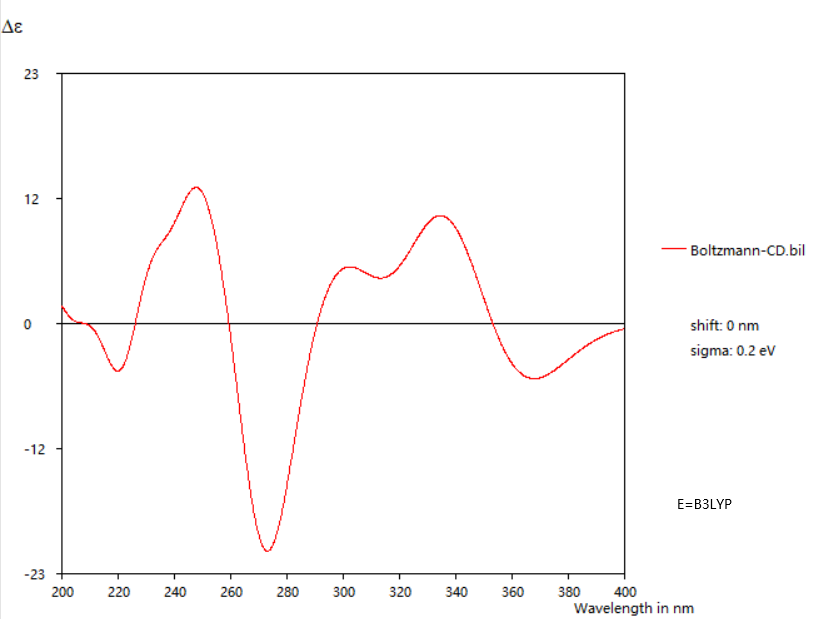


**Figure S5.** X-ray diffraction of compound **2**.


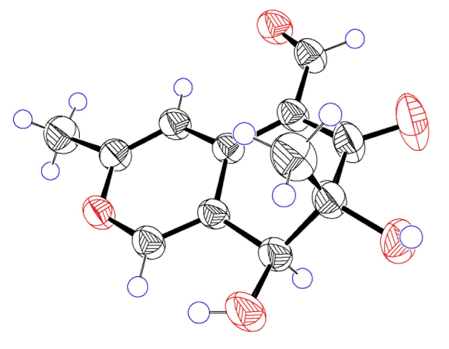


Flack parameter: 0.03(15)

**Figure S6.**^1^H NMR spectrum of compound **3** (500 MHz, CD_3_OD).


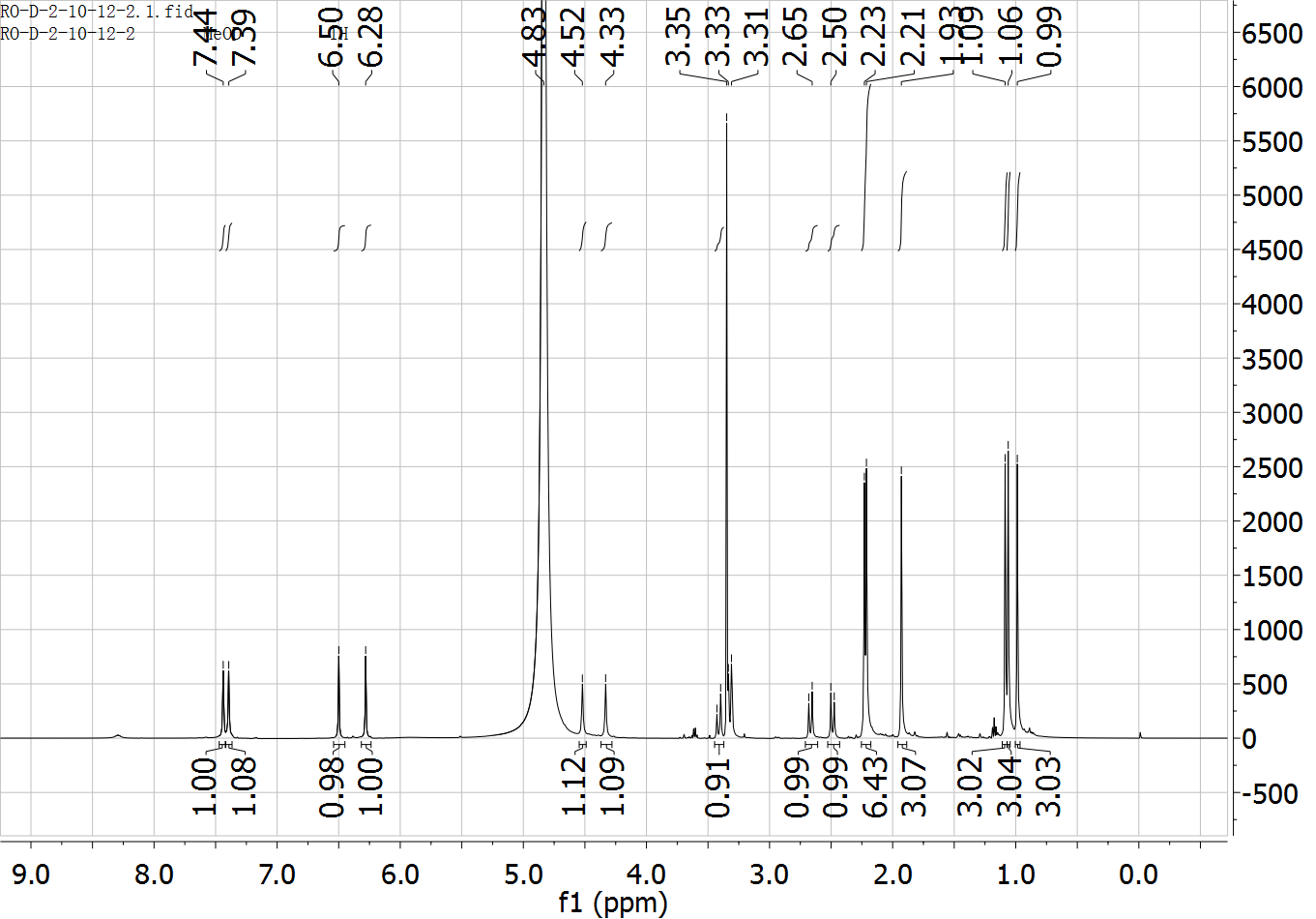


**Figure S7.**^13^C NMR spectrum of compound **3** (125 MHz, CD_3_OD).


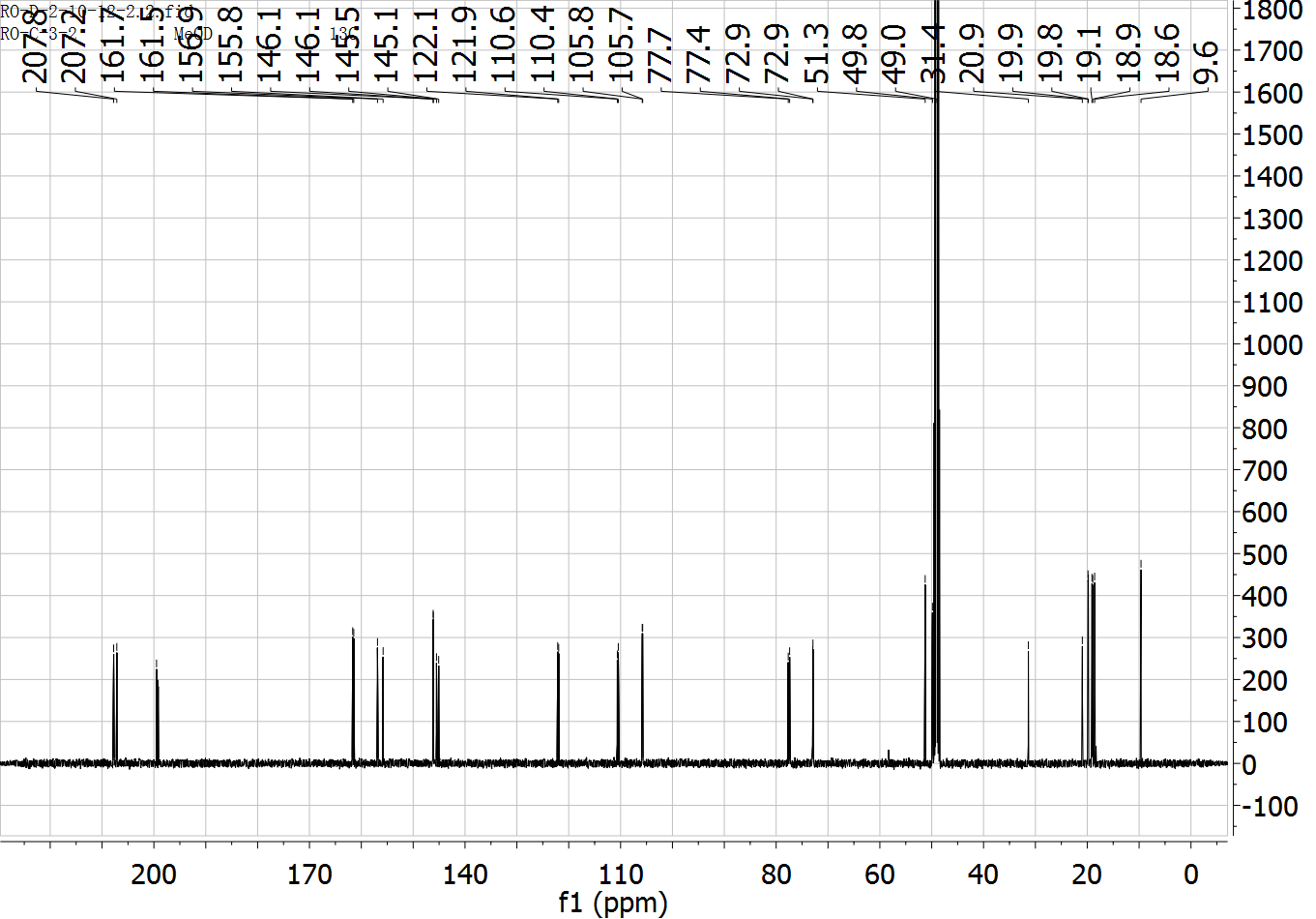


**Figure S8.** HSQC spectrum of compound **3** (500 MHz, CD_3_OD).


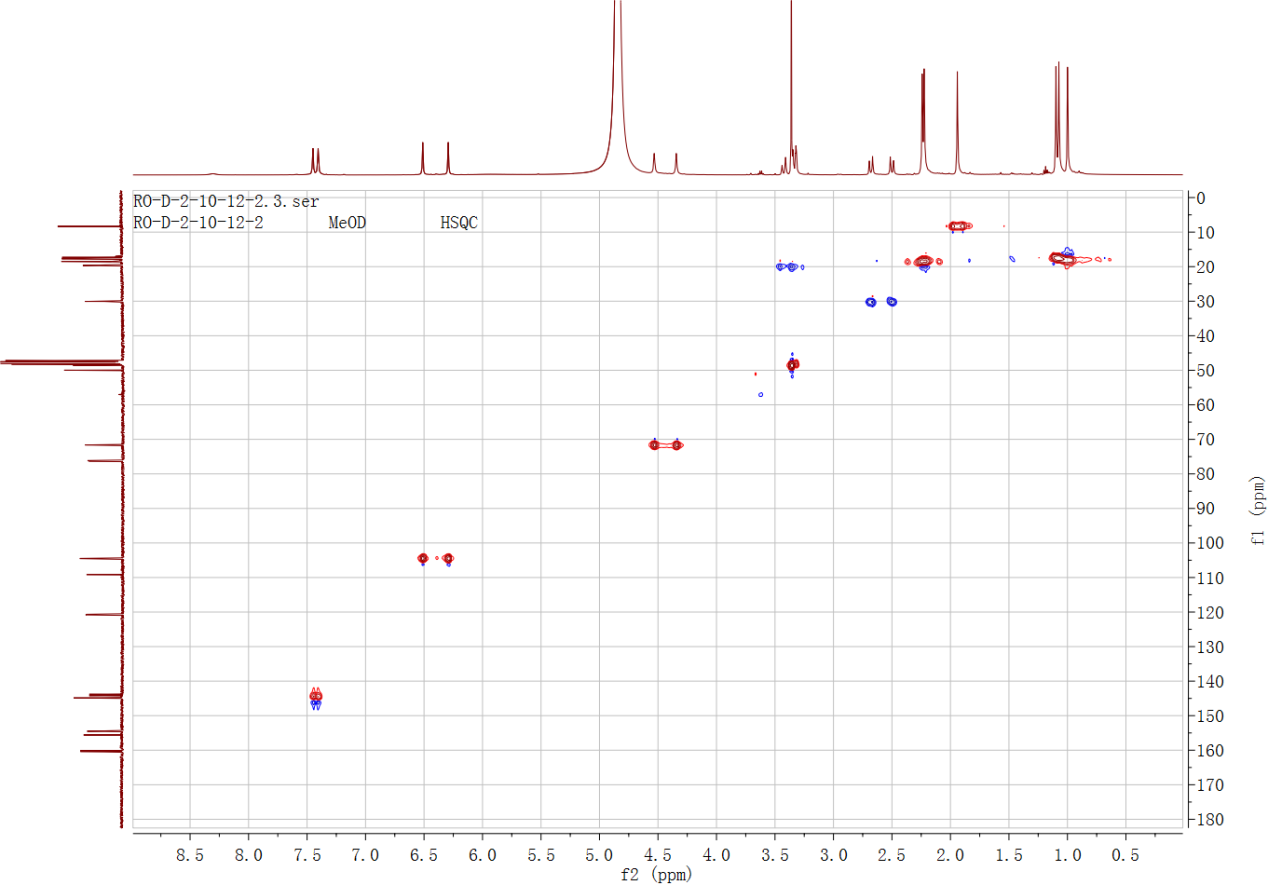


**Figure S9.** HMBC spectrum of compound **3** (500 MHz, CD_3_OD).


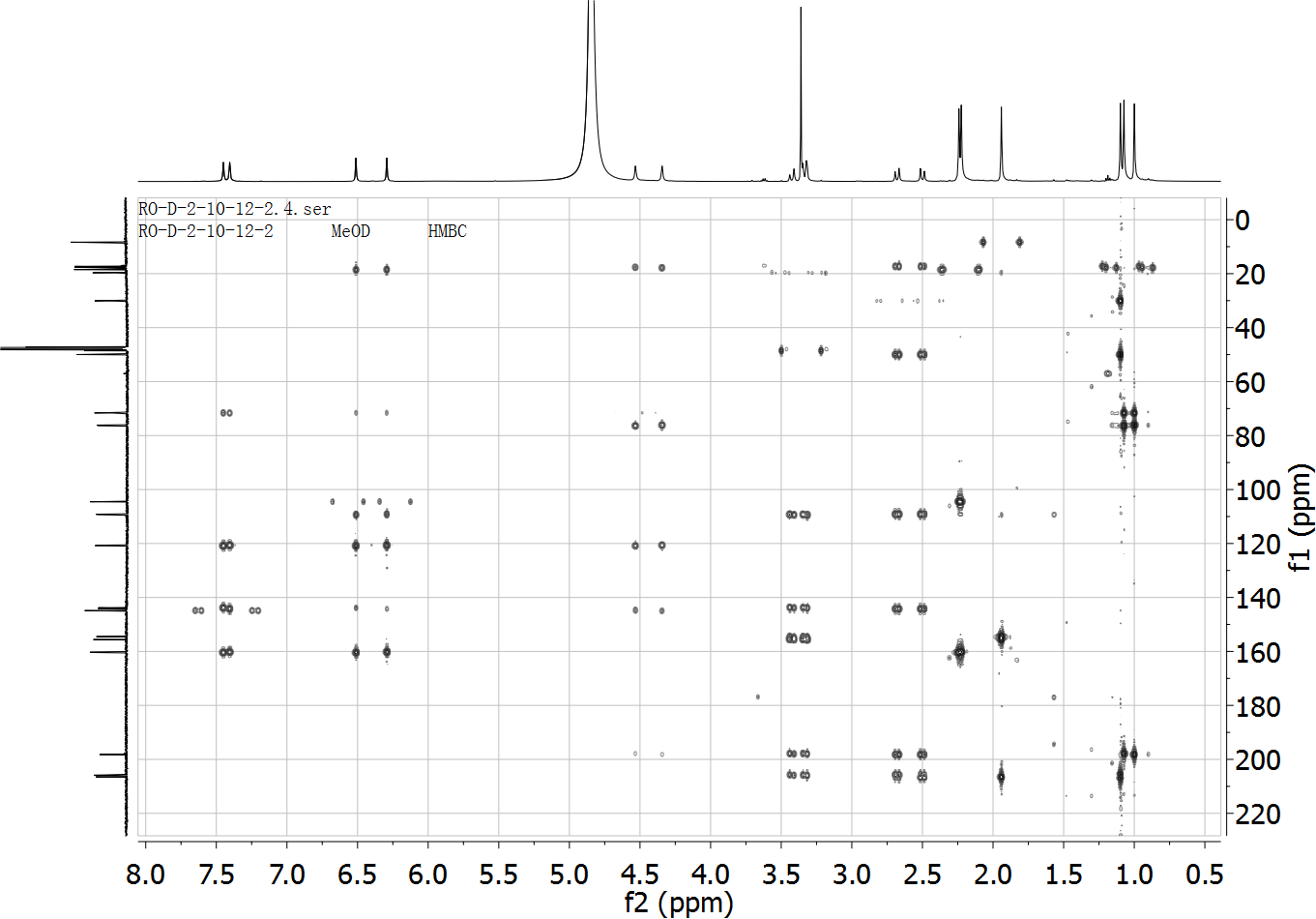


**Figure S10.** ROESY spectrum of compound **3** (500 MHz, CD_3_OD).


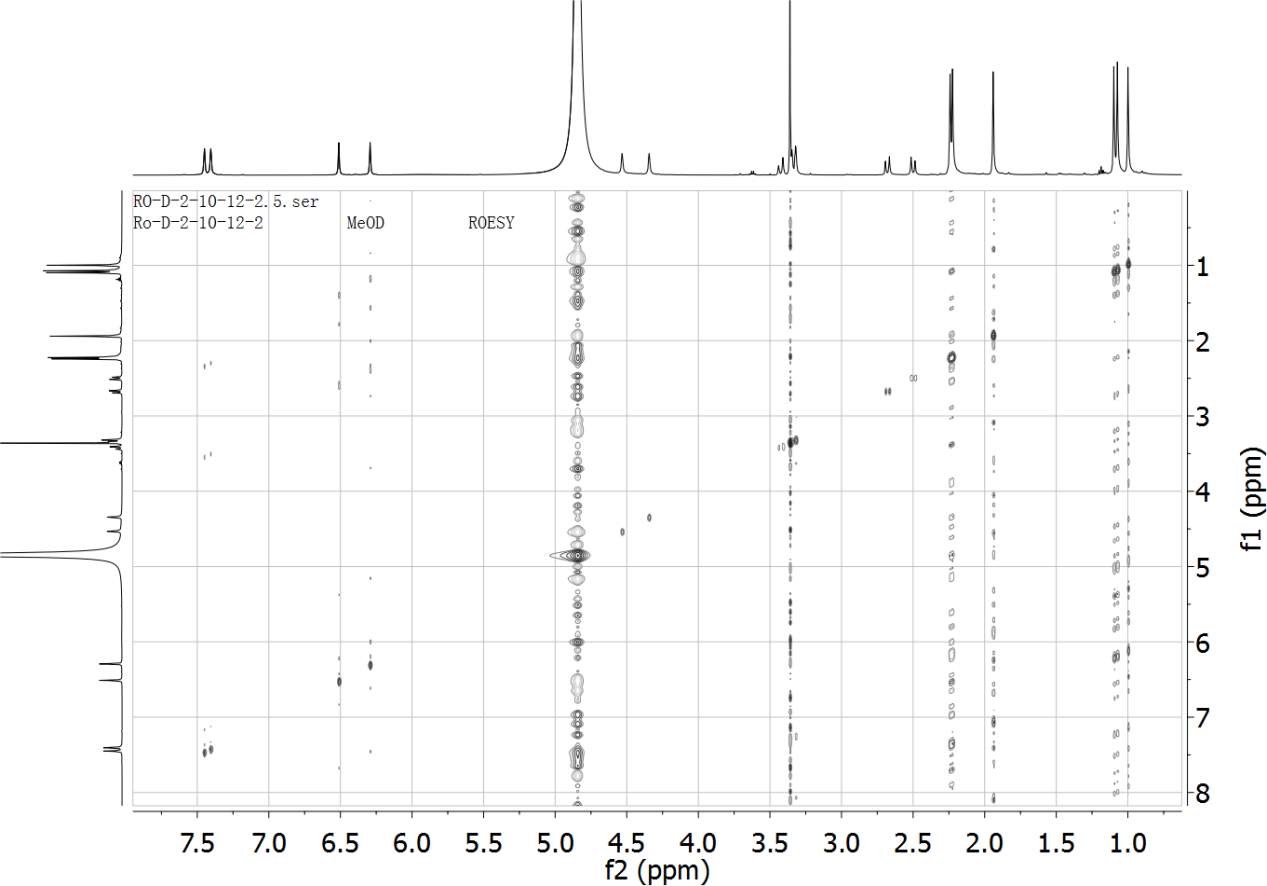


**Figure S11.** IR spectrum of compound **3**.

**
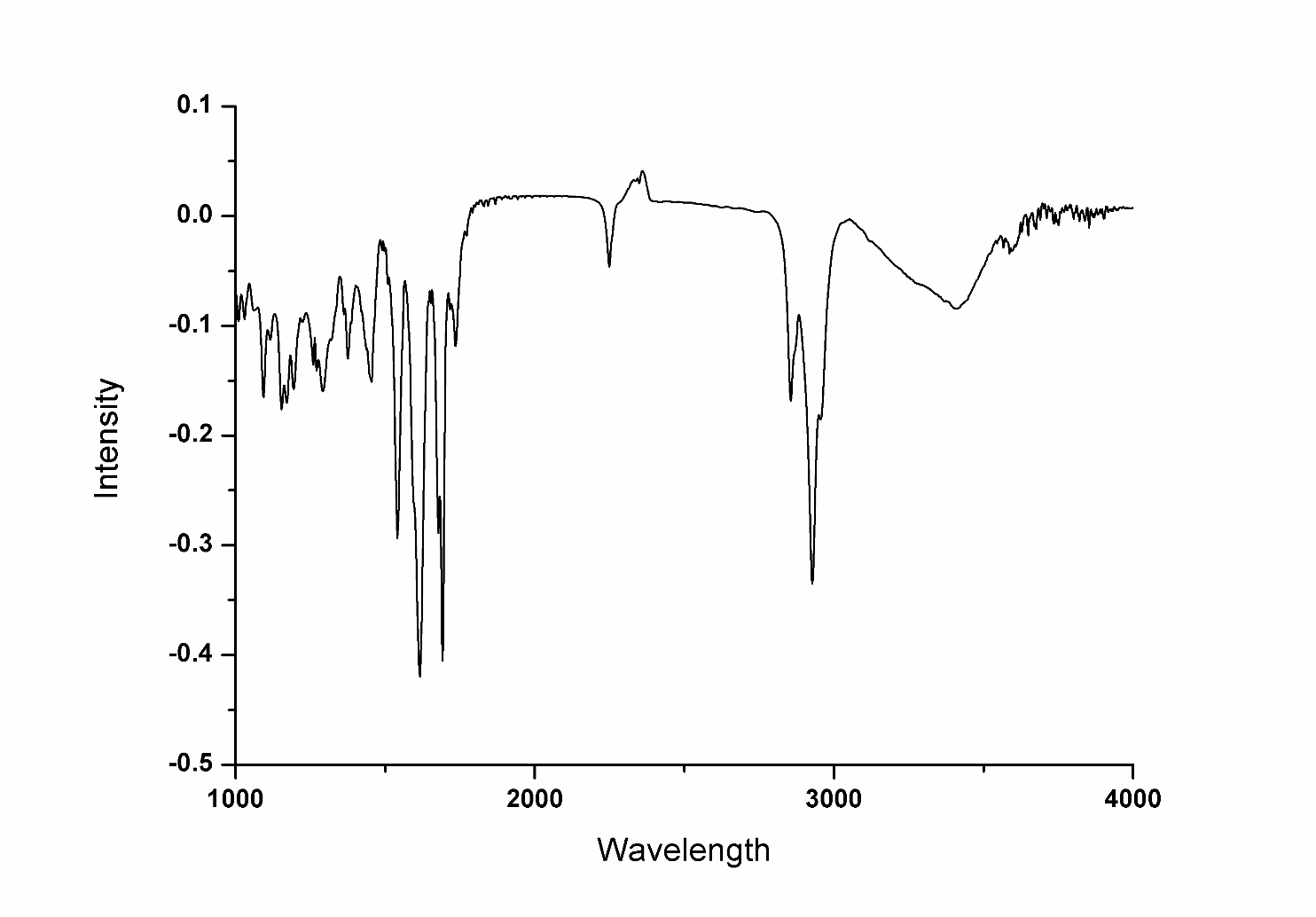
**

**Figure S12.** Positive ESI-HRMS spectrum of compound **3**.


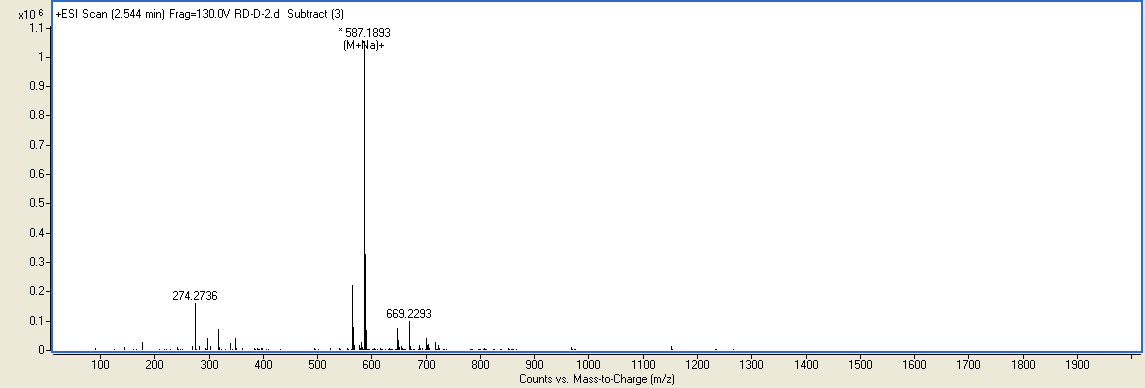


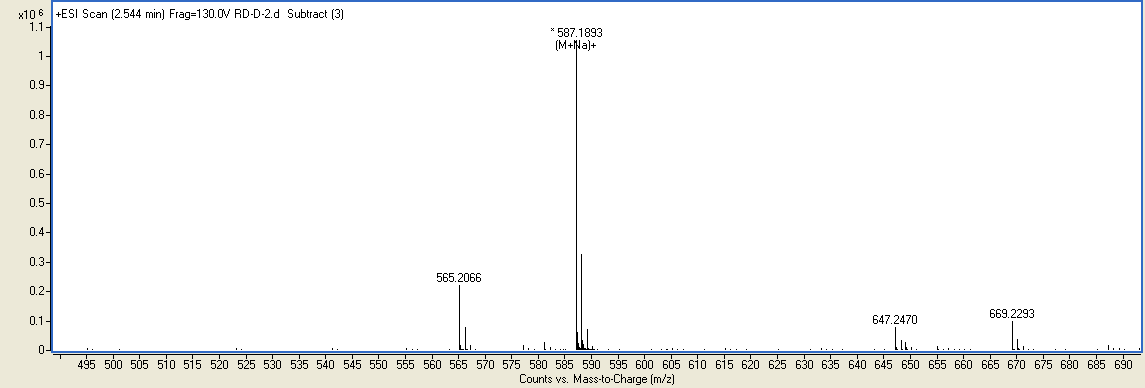


*m/z* 565.2066, [M+H]^+^; calcd. for C_31_H_33_O_10_, *m/z* 565.2068, Δ -0.3539 ppm

*m/z* 587.1893, [M+Na]^+^; calcd. for C_31_H_32_O_10_Na, *m/z* 587.1888, Δ 0.8515 ppm

**Figure S13.** The individual ECD spectra of each conformation for (7*R*,8*S*,7'*R*,8'*S*,15*R*)-**3** at the TDDFT-B3LYP/6-311G(2d,p)/SMD (methanol) level.


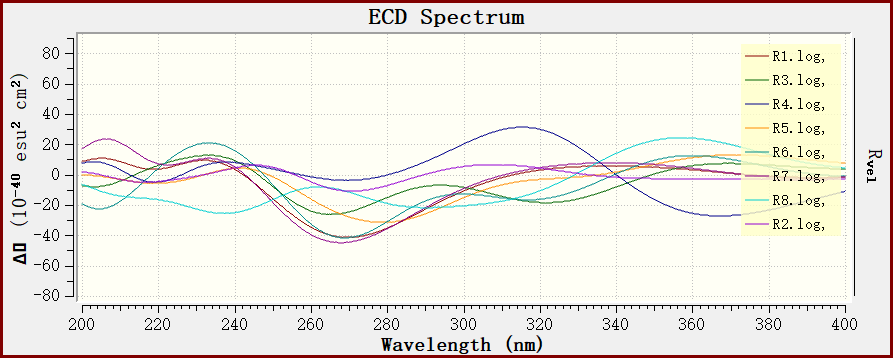


**Figure S14.** The individual ECD spectra of each conformation for (7*R*,8*S*,7'*R*,8'*S*,15*S*)-**3** at the TDDFT-B3LYP/6-311G(2d,p)/SMD(methanol) level.


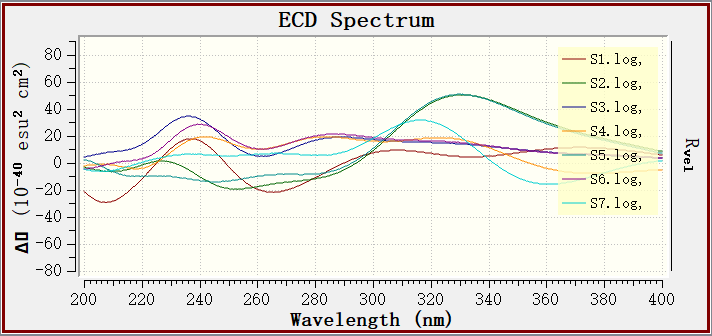


**Figure S15.** Fungal ITS sequence and phylogenetic tree.

ITS sequence:

ccgtaggtgaacctgcggaaggatcattacaagttgaaacggttgccctcgcggtgaccggttcttcaaacctctgcgtaccaaacctttcagttgcctccggcggccctgggccgacgcggcgcgcgacctccccttcgcgggggggccgctcctcgcggcggaccacccgccgggcggtcataaacaaaacctttttgtcgagatggcatcgtctaatttcttcataacaaaatatgaaatacaacttttaacaatggatctcttggctccggcatcgatgaagaacgcagcgaaatgcgataactagtgtgaattgcagatttcagtgaatcatcgagtctttgaacgcacattgcgcctcttggtattcctcgaggcatgcctgttcgagcgtcgttacgcccctcaagcgcaagcttggtgttggggatcgcccctgagatacggcggcggcccttaaatgcatcggcggtgctggtgtcagcccggagcgcagcagacatgcggcttccaggcgaccacgcgcccgccggacaacgacccgactttcaaacgtcgacctcggatcaggtagggatacccgctgaacttaagcatatcaataa

Phylogenetic tree:

**

**

**Figure S16.** Total conformer population of (7*R*,8*S*,7'*R*,8'*S*,15*R*)-**3**. The energies are computed at the B3LYP-D3BJ/6-311G(2d,p) level. P stands for the value of the conformer population.


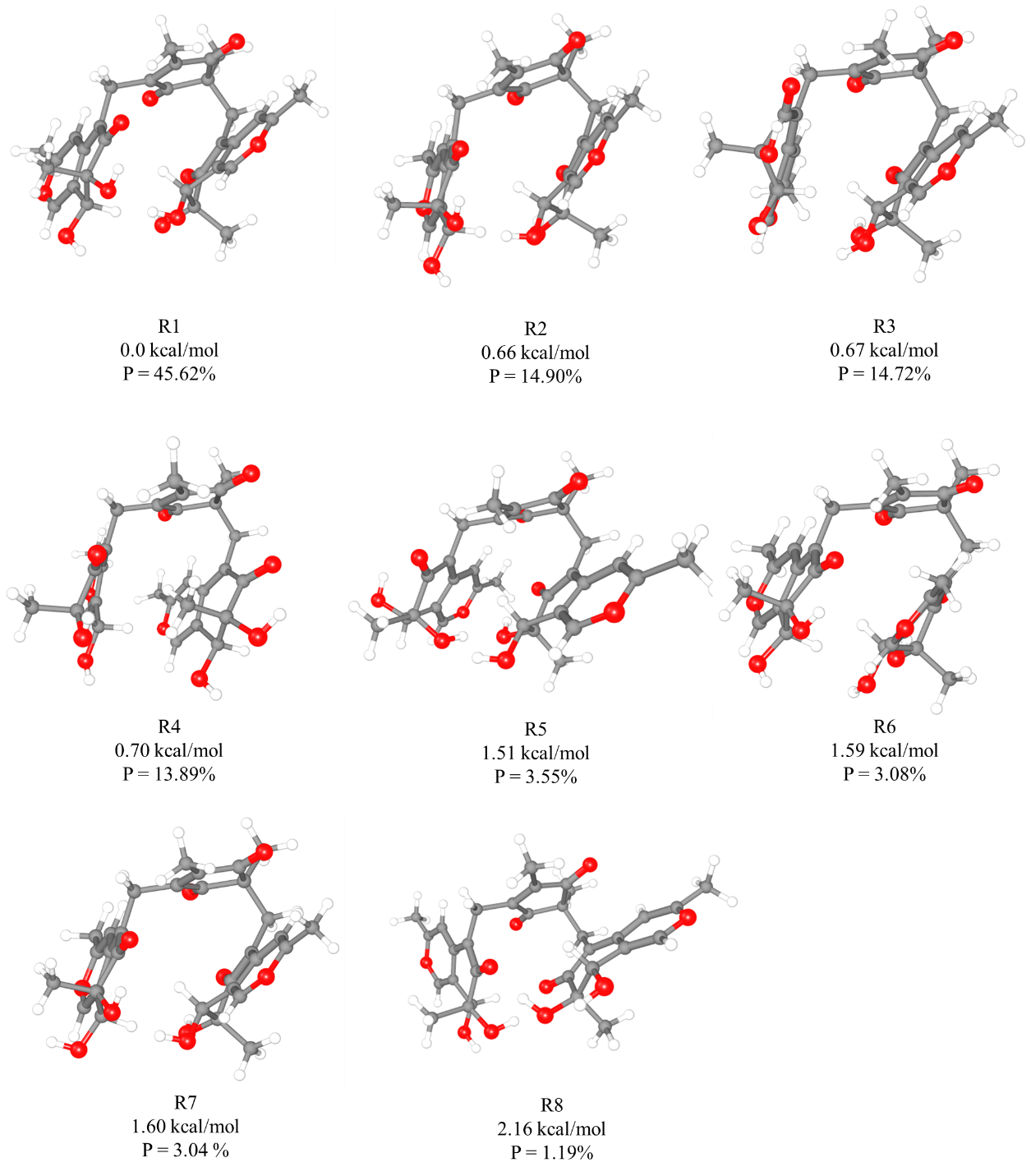


**Figure S17.** Total conformer population of (7*R*,8*S*,7'*R*,8'*S*,15*S*)-**3**. The energies are computed at the B3LYP-D3BJ/6-311G(2d,p) level. P stands for the value of the conformer population.

**
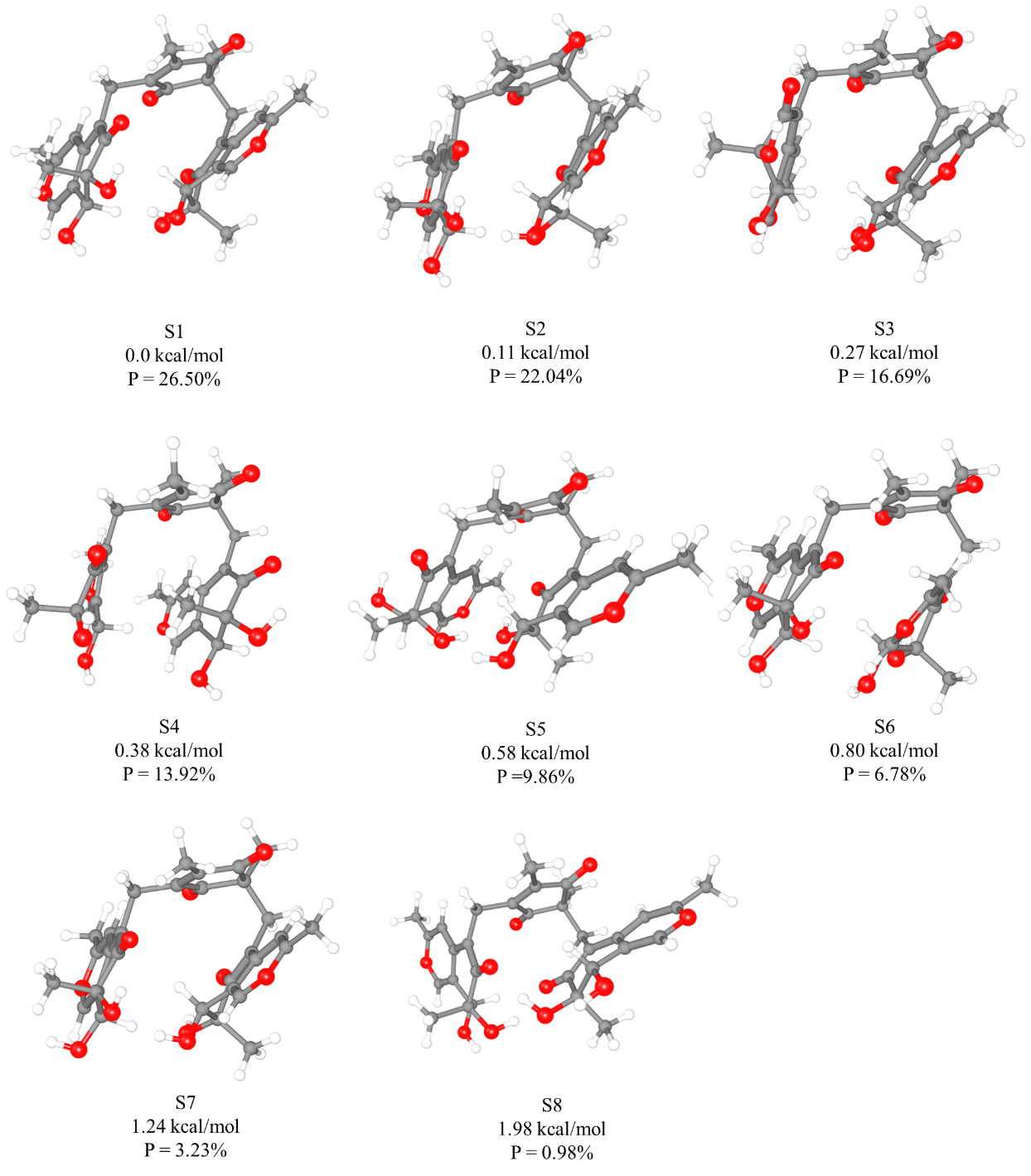
**

**Table S1.** The calculated relative energies (△*E,* kcal/mol) at the MMFF94S level. The energy threshold is 10 kcal/mol.

| **15*R*-3**  **Conformers** | **MMFF94S**  **E(kcal/mol)** |  | **15*S*-3**  **Conformers** | **MMFF94S**  **E(kcal/mol)** |
| --- | --- | --- | --- | --- |
| R_1 | 0 |  | S_1 | 0 |
| R_2 | 1.57 |  | S_2 | 0.88 |
| R_3 | 1.86 |  | S_3 | 2.09 |
| R_4 | 1.96 |  | S_4 | 2.25 |
| R_5 | 2.28 |  | S_5 | 2.84 |
| R_6 | 2.44 |  | S_6 | 2.91 |
| R_7 | 2.49 |  | S_7 | 2.96 |
| R_8 | 2.5 |  | S_8 | 3.8 |
| R_9 | 2.67 |  | S_9 | 3.87 |
| R_10 | 2.77 |  | S_10 | 3.88 |
| R_11 | 2.92 |  | S_11 | 4.08 |
| R_12 | 3.04 |  | S_12 | 4.52 |
| R_13 | 3.2 |  | S_13 | 4.54 |
| R_14 | 3.63 |  | S_14 | 4.57 |
| R_15 | 3.66 |  | S_15 | 4.7 |
| R_16 | 3.7 |  | S_16 | 4.88 |
| R_17 | 3.83 |  | S_17 | 4.9 |
| R_18 | 3.83 |  | S_18 | 5.06 |
| R_19 | 3.87 |  | S_19 | 5.13 |
| R_20 | 4.01 |  | S_20 | 5.44 |
| R_21 | 4.05 |  | S_21 | 5.72 |
| R_22 | 4.1 |  | S_22 | 5.83 |
| R_23 | 4.34 |  | S_23 | 5.91 |
| R_24 | 4.4 |  | S_24 | 5.98 |
| R_25 | 4.62 |  | S_25 | 6.02 |
| R_26 | 4.63 |  | S_26 | 6.17 |
| R_27 | 4.9 |  | S_27 | 6.19 |
| R_28 | 4.93 |  | S_28 | 6.21 |
| R_29 | 4.94 |  | S_29 | 6.25 |
| R_30 | 4.94 |  | S_30 | 6.33 |
| R_31 | 4.97 |  | S_31 | 6.48 |
| R_32 | 4.99 |  | S_32 | 6.61 |
| R_33 | 5.04 |  | S_33 | 6.65 |
| R_34 | 5.17 |  | S_34 | 6.77 |
| R_35 | 5.21 |  | S_35 | 6.93 |
| R_36 | 5.34 |  | S_36 | 7.09 |
| R_37 | 5.46 |  | S_37 | 7.1 |
| R_38 | 5.68 |  | S_38 | 7.16 |
| R_39 | 5.68 |  | S_39 | 7.52 |
| R_40 | 5.87 |  | S_40 | 7.57 |
| R_41 | 6.02 |  | S_41 | 7.61 |
| R_42 | 6.4 |  | S_42 | 7.87 |
| R_43 | 6.56 |  | S_43 | 7.9 |
| R_44 | 6.62 |  | S_44 | 8.23 |
| R_45 | 6.68 |  | S_45 | 8.34 |
| R_46 | 7.12 |  | S_46 | 8.44 |
| R_47 | 7.33 |  | S_47 | 8.63 |
| R_48 | 7.44 |  | S_48 | 8.67 |
| R_49 | 7.61 |  | S_49 | 8.8 |
| R_50 | 7.79 |  | S_50 | 9.06 |
| R_51 | 8.04 |  | S_51 | 9.08 |
| R_52 | 8.25 |  | S_52 | 9.27 |
| R_53 | 8.71 |  | S_53 | 9.52 |
| R_54 | 8.88 |  | S_54 | 9.59 |
| R_55 | 8.95 |  | S_55 | 9.62 |
| R_56 | 8.97 |  | S_56 | 10.27 |
| R_57 | 9.17 |  | S_57 | 10.49 |
| R_58 | 9.31 |  | S_58 | 10.54 |
| R_59 | 9.32 |  | S_59 | 10.81 |
| R_60 | 9.91 |  | S_60 | 10.97 |
| R_61 | 10.06 |  |  |  |

**Table S2.** The Cartesian coordinates of conformers for (7*R*,8*S*,7'*R*,8'*S*,15*R*)-**3** and (7*R*,8*S*,7'*R*,8'*S*,15*S*)-**3** at the B3LYP-D3BJ/6-311G(2d,p) level.

**(7*R*,8*S*,7'*R*,8'*S*,15*R*)-3:**

**R1**

----------------------------------------------------------------------------------

Coordinates (Angstroms)

X Y Z

-----------------------------------------------------------------------------------

C 4.377672000 2.073926000 0.472419000

O 5.107614000 1.000814000 0.870388000

C 4.615218000 -0.239834000 0.636420000

C 3.481893000 -0.471747000 -0.043468000

C 2.737616000 0.636405000 -0.602309000

C 3.232268000 1.928361000 -0.226077000

C 2.921248000 -1.858229000 -0.148272000

C 2.252401000 -2.062526000 -1.502195000

C 1.301421000 -0.895826000 -1.829941000

C 1.647435000 0.434786000 -1.434885000

C 4.992514000 3.357788000 0.893836000

O 0.299985000 -1.186837000 -2.498951000

O 3.940911000 -2.814015000 0.112764000

C 3.263169000 -2.203754000 -2.640019000

O 1.453713000 -3.252882000 -1.417511000

C -0.650344000 0.185824000 2.137389000

C -0.258132000 -1.302673000 2.252959000

C -0.600587000 -1.966614000 0.919313000

C -2.063315000 -1.767222000 0.638904000

C -2.625304000 -0.453649000 0.868896000

C -1.882356000 0.541266000 1.481489000

C -2.814545000 -2.738635000 0.104486000

O -4.111920000 -2.567307000 -0.257524000

C -4.661932000 -1.331631000 -0.130477000

C -3.969869000 -0.297514000 0.392303000

O 0.110838000 1.000841000 2.667139000

C -0.969612000 -1.960282000 3.436838000

O -0.215116000 -3.323394000 0.868161000

O 1.143669000 -1.360347000 2.464165000

C -6.062779000 -1.289637000 -0.621235000

C -0.446701000 1.898478000 -1.404061000

C -0.507761000 2.698606000 -0.152203000

C -1.968677000 2.842975000 0.279913000

C -2.685673000 2.302517000 -0.951763000

C -1.698747000 1.673497000 -1.863366000

C -2.328557000 4.304579000 0.542015000

O -3.876388000 2.399038000 -1.172654000

C -2.130729000 0.980577000 -3.105003000

O 0.440042000 3.228313000 0.386802000

C 0.852737000 1.559642000 -2.070475000

C -2.301646000 1.986134000 1.539921000

H 5.233141000 -1.012301000 1.065487000

H 2.676049000 2.817094000 -0.473243000

H 2.150557000 -1.930841000 0.624464000

H 5.087710000 3.390062000 1.982444000

H 5.996630000 3.455076000 0.472001000

H 4.383973000 4.197989000 0.565161000

H 3.502680000 -3.639489000 0.354766000

H 3.898482000 -1.319287000 -2.716054000

H 3.894046000 -3.071837000 -2.453539000

H 2.737615000 -2.338557000 -3.586615000

H 0.717532000 -3.078700000 -2.035363000

H -0.065520000 -1.396530000 0.154344000

H -2.459138000 -3.736282000 -0.099212000

H -4.469224000 0.655642000 0.453685000

H -0.699682000 -1.438488000 4.356341000

H -2.055212000 -1.943484000 3.330450000

H -0.640273000 -2.997387000 3.513159000

H 0.426476000 -3.411571000 0.138469000

H 1.371724000 -0.455027000 2.749011000

H -6.474449000 -0.287867000 -0.515315000

H -6.101518000 -1.581165000 -1.674308000

H -6.683994000 -1.994118000 -0.061525000

H -3.388262000 4.391225000 0.786339000

H -1.739249000 4.688028000 1.375806000

H -2.128114000 4.925260000 -0.334201000

H -3.141802000 0.590563000 -2.981527000

H -2.157467000 1.681679000 -3.946111000

H -1.448476000 0.168498000 -3.350312000

H 0.647334000 1.280298000 -3.104206000

H 1.458898000 2.467175000 -2.094183000

H -3.376117000 2.081574000 1.712777000

H -1.791086000 2.455464000 2.380681000

-----------------------------------------------------------------------------------

**R2**

----------------------------------------------------------------------------------

Coordinates (Angstroms)

X Y Z

-----------------------------------------------------------------------------------

C 0.680390000 2.626701000 -2.148315000

O 1.511094000 3.312253000 -1.316301000

C 2.309973000 2.611973000 -0.472354000

C 2.379500000 1.273613000 -0.467645000

C 1.595873000 0.500742000 -1.406664000

C 0.695019000 1.278039000 -2.207119000

C 3.182742000 0.527226000 0.549546000

C 3.878973000 -0.655906000 -0.111286000

C 2.814878000 -1.521536000 -0.810909000

C 1.721847000 -0.876465000 -1.485175000

C -0.228301000 3.528243000 -2.897256000

O 3.013856000 -2.741781000 -0.806458000

O 4.074190000 1.404116000 1.213721000

C 4.944487000 -0.232434000 -1.124614000

O 4.475164000 -1.425384000 0.924240000

C -2.850908000 1.472167000 0.122411000

C -2.197949000 2.672932000 0.829200000

C -0.725633000 2.345612000 1.040295000

C -0.579790000 1.060724000 1.792287000

C -1.482170000 -0.026757000 1.467882000

C -2.526961000 0.140266000 0.578881000

C 0.413414000 0.875515000 2.671515000

O 0.649750000 -0.308028000 3.296083000

C -0.146924000 -1.368929000 3.000531000

C -1.173166000 -1.262946000 2.131060000

O -3.675354000 1.725851000 -0.757674000

C -2.936925000 2.979743000 2.132868000

O -0.004490000 3.382675000 1.681482000

O -2.272420000 3.801157000 -0.034195000

C 0.258095000 -2.594127000 3.734264000

C -0.468415000 -2.110504000 -1.608576000

C -1.737605000 -1.361004000 -1.830513000

C -2.838694000 -1.960996000 -0.957535000

C -2.098914000 -3.113969000 -0.295919000

C -0.687358000 -3.131363000 -0.748211000

C -3.973118000 -2.509043000 -1.833028000

O -2.588914000 -3.908310000 0.482847000

C 0.235980000 -4.183154000 -0.255644000

O -1.888510000 -0.464718000 -2.634189000

C 0.802145000 -1.745236000 -2.320572000

C -3.422500000 -0.970674000 0.094202000

H 2.857521000 3.253951000 0.198130000

H -0.013869000 0.796503000 -2.861999000

H 2.480198000 0.092415000 1.274063000

H -0.846316000 2.957119000 -3.587438000

H -0.877946000 4.048679000 -2.188570000

H 0.341322000 4.277781000 -3.452059000

H 4.536737000 0.879621000 1.880967000

H 5.416380000 -1.122559000 -1.543159000

H 4.522462000 0.355885000 -1.940969000

H 5.704111000 0.364918000 -0.619839000

H 4.331874000 -2.348457000 0.648991000

H -0.320999000 2.172433000 0.039364000

H 1.129021000 1.631912000 2.951653000

H -1.778808000 -2.140061000 1.972362000

H -3.981251000 3.206283000 1.913092000

H -2.900598000 2.142793000 2.831726000

H -2.480508000 3.849582000 2.606127000

H -0.067237000 4.158112000 1.107978000

H -2.967343000 3.560847000 -0.675372000

H 1.281898000 -2.869665000 3.466207000

H 0.237255000 -2.416846000 4.812782000

H -0.408819000 -3.420294000 3.495519000

H -4.422136000 -1.692869000 -2.399970000

H -3.607303000 -3.261357000 -2.535663000

H -4.736923000 -2.969347000 -1.204275000

H -0.228989000 -5.166180000 -0.364287000

H 1.197212000 -4.158204000 -0.760712000

H 0.413872000 -4.043209000 0.815613000

H 1.342913000 -2.657329000 -2.572709000

H 0.539838000 -1.241010000 -3.251942000

H -3.786692000 -1.578240000 0.927627000

H -4.289720000 -0.496566000 -0.361325000

-----------------------------------------------------------------------------------

**R3**

----------------------------------------------------------------------------------

Coordinates (Angstroms)

X Y Z

-----------------------------------------------------------------------------------

C 2.678600000 2.602035000 -0.739721000

O 3.627291000 2.273937000 0.173083000

C 3.736368000 0.980781000 0.555417000

C 3.023521000 -0.014423000 0.006531000

C 2.094823000 0.268555000 -1.067152000

C 1.926101000 1.662363000 -1.350030000

C 3.088115000 -1.414721000 0.530742000

C 3.046340000 -2.403145000 -0.628251000

C 1.800857000 -2.112394000 -1.486993000

C 1.424957000 -0.747481000 -1.730647000

C 2.596969000 4.066261000 -0.968489000

O 1.238090000 -3.095390000 -1.984286000

O 4.227095000 -1.573179000 1.359332000

C 4.296283000 -2.337075000 -1.509826000

O 2.919615000 -3.704130000 -0.071629000

C -0.948402000 2.185665000 1.046646000

C -0.005112000 1.837777000 2.214338000

C 0.328740000 0.354363000 2.102797000

C -0.932306000 -0.446000000 2.141062000

C -2.057622000 0.043315000 1.372963000

C -2.010966000 1.265761000 0.722508000

C -1.011051000 -1.616229000 2.789281000

O -2.114664000 -2.405808000 2.771449000

C -3.183729000 -2.011271000 2.032817000

C -3.180458000 -0.849048000 1.345917000

O -0.770213000 3.273976000 0.493208000

C -0.649143000 2.208956000 3.551329000

O 1.238572000 -0.098444000 3.087713000

O 1.199931000 2.571203000 2.054717000

C -4.297687000 -2.990657000 2.088510000

C -1.002571000 -0.201497000 -2.329959000

C -1.528481000 1.189031000 -2.237786000

C -2.959101000 1.156676000 -1.705771000

C -3.244720000 -0.338951000 -1.741045000

C -1.993029000 -1.077509000 -2.041191000

C -3.913685000 1.922143000 -2.622974000

O -4.335818000 -0.852123000 -1.590139000

C -1.980786000 -2.561000000 -2.047883000

O -0.940237000 2.186403000 -2.599481000

C 0.391726000 -0.487472000 -2.811009000

C -3.056215000 1.729586000 -0.258127000

H 4.458200000 0.847475000 1.345453000

H 1.179180000 1.994497000 -2.053816000

H 2.178459000 -1.594761000 1.120420000

H 2.443944000 4.588785000 -0.020936000

H 3.530770000 4.438151000 -1.399667000

H 1.773559000 4.299081000 -1.640239000

H 4.182992000 -2.466504000 1.725253000

H 4.211185000 -3.074189000 -2.309567000

H 4.432324000 -1.350452000 -1.955783000

H 5.173132000 -2.571008000 -0.905820000

H 2.289425000 -4.155468000 -0.661469000

H 0.762105000 0.219505000 1.106056000

H -0.211857000 -2.046856000 3.371347000

H -4.068856000 -0.601023000 0.789033000

H -1.580219000 1.668532000 3.727410000

H 0.046700000 1.978114000 4.358722000

H -0.859593000 3.279411000 3.565028000

H 2.064054000 0.385233000 2.949308000

H 0.957915000 3.288400000 1.440421000

H -3.955689000 -3.968509000 1.739031000

H -4.647961000 -3.112530000 3.117053000

H -5.125869000 -2.660452000 1.464595000

H -3.627210000 2.973676000 -2.661334000

H -3.893331000 1.521834000 -3.638925000

H -4.934612000 1.845326000 -2.246271000

H -1.013846000 -2.961129000 -2.342028000

H -2.220456000 -2.932702000 -1.046410000

H -2.764866000 -2.938234000 -2.710155000

H 0.361640000 -1.370963000 -3.449087000

H 0.706124000 0.357517000 -3.427041000

H -4.064718000 1.509362000 0.101491000

H -2.969945000 2.811948000 -0.347853000

-----------------------------------------------------------------------------------

**R4**

----------------------------------------------------------------------------------

Coordinates (Angstroms)

X Y Z

-----------------------------------------------------------------------------------

C 3.338264000 2.437389000 -0.359960000

O 4.155448000 1.560391000 0.284039000

C 3.827804000 0.242859000 0.276060000

C 2.785971000 -0.251374000 -0.405625000

C 1.943480000 0.628198000 -1.187926000

C 2.252526000 2.021825000 -1.043454000

C 2.414225000 -1.699179000 -0.343780000

C 2.048648000 -2.183350000 -1.744426000

C 0.921412000 -1.287669000 -2.290400000

C 0.949268000 0.122736000 -2.009076000

C 3.803770000 3.840812000 -0.221430000

O 0.076883000 -1.837574000 -3.005069000

O 3.456083000 -2.451321000 0.252939000

C 3.238381000 -2.157443000 -2.707360000

O 1.559330000 -3.512278000 -1.630695000

C -2.799583000 -0.898882000 1.329238000

C -1.978512000 -2.196198000 1.333006000

C -0.976089000 -2.135874000 2.481516000

C -0.168854000 -0.872625000 2.421422000

C -0.816465000 0.351196000 1.988131000

C -2.109245000 0.358545000 1.498983000

C 1.103730000 -0.849191000 2.842656000

O 1.844124000 0.284606000 2.937085000

C 1.255184000 1.468552000 2.617870000

C -0.008026000 1.528975000 2.153152000

O -4.009984000 -1.001967000 1.122730000

C -1.309435000 -2.355919000 -0.030407000

O -0.110864000 -3.259597000 2.516360000

O -2.845503000 -3.293044000 1.577023000

C 2.152353000 2.624865000 2.870616000

C -1.351033000 1.161320000 -1.950201000

C -1.478911000 2.172285000 -0.872688000

C -2.882206000 2.112219000 -0.275438000

C -3.559092000 1.118999000 -1.216424000

C -2.539097000 0.539974000 -2.133564000

C -3.566899000 3.478931000 -0.354649000

O -4.747063000 0.892865000 -1.272919000

C -2.916181000 -0.502455000 -3.120039000

O -0.620271000 2.969774000 -0.547441000

C -0.073793000 0.983357000 -2.722296000

C -2.878149000 1.619184000 1.199473000

H 4.496902000 -0.346958000 0.881596000

H 1.626837000 2.771061000 -1.498120000

H 1.506471000 -1.785398000 0.263709000

H 3.894161000 4.113411000 0.832567000

H 4.791374000 3.957791000 -0.676404000

H 3.107828000 4.522102000 -0.707332000

H 3.127179000 -3.356013000 0.339773000

H 2.918407000 -2.518620000 -3.685706000

H 3.651922000 -1.154216000 -2.822349000

H 4.019933000 -2.815289000 -2.326480000

H 0.783753000 -3.536718000 -2.219381000

H -1.575707000 -2.101619000 3.403308000

H 1.657054000 -1.720355000 3.155399000

H -0.389259000 2.497595000 1.872217000

H -2.074843000 -2.439618000 -0.801700000

H -0.678360000 -1.501136000 -0.261848000

H -0.696327000 -3.255378000 -0.041159000

H -0.673582000 -4.041505000 2.596797000

H -3.735623000 -2.934874000 1.403883000

H 3.149127000 2.432776000 2.473544000

H 1.746663000 3.527022000 2.417391000

H 2.255811000 2.787940000 3.947840000

H -3.019480000 4.206839000 0.245832000

H -3.607432000 3.843077000 -1.383599000

H -4.588192000 3.403667000 0.021110000

H -2.044408000 -1.041902000 -3.482739000

H -3.621078000 -1.202802000 -2.667685000

H -3.436295000 -0.044138000 -3.968330000

H -0.303604000 0.517444000 -3.679868000

H 0.340520000 1.972407000 -2.924638000

H -3.922941000 1.452878000 1.468831000

H -2.512949000 2.443297000 1.813267000

-----------------------------------------------------------------------------------

**R5**

----------------------------------------------------------------------------------

Coordinates (Angstroms)

X Y Z

-----------------------------------------------------------------------------------

C -3.502575000 2.190451000 -1.366353000

O -4.378841000 1.294389000 -1.905644000

C -4.531275000 0.097537000 -1.290442000

C -3.824959000 -0.283162000 -0.216711000

C -2.824039000 0.602854000 0.341208000

C -2.769267000 1.897033000 -0.274164000

C -4.126527000 -1.573456000 0.495664000

C -2.819945000 -2.214596000 0.983404000

C -1.996593000 -1.181017000 1.806036000

C -2.007539000 0.204924000 1.383901000

C -3.443298000 3.452031000 -2.144808000

O -1.330799000 -1.578559000 2.762147000

O -5.044545000 -1.351706000 1.569187000

C -3.077855000 -3.469420000 1.789562000

O -2.064768000 -2.616374000 -0.168317000

C 0.718720000 0.548499000 -1.629960000

C 0.296344000 -0.882881000 -2.002708000

C 0.830647000 -1.813414000 -0.907050000

C 2.321347000 -1.647206000 -0.838715000

C 2.859735000 -0.305206000 -0.867722000

C 2.036576000 0.782727000 -1.120920000

C 3.135742000 -2.696946000 -0.661239000

O 4.476283000 -2.578339000 -0.495551000

C 5.016610000 -1.333668000 -0.426927000

C 4.262227000 -0.225294000 -0.582767000

O -0.116752000 1.441615000 -1.818498000

C 0.793313000 -1.267596000 -3.394185000

O 0.482419000 -3.166648000 -1.091396000

O -1.132947000 -0.900469000 -2.009241000

C 6.478458000 -1.372754000 -0.171873000

C 0.242791000 1.469021000 1.732171000

C 0.583011000 2.664184000 0.907941000

C 2.084376000 2.670222000 0.621180000

C 2.559366000 1.602427000 1.598334000

C 1.386345000 0.852947000 2.115118000

C 2.701430000 4.038039000 0.894412000

O 3.709682000 1.407440000 1.931759000

C 1.592461000 -0.334999000 2.978742000

O -0.187585000 3.550134000 0.598879000

C -1.165520000 1.188531000 2.180842000

C 2.415566000 2.210367000 -0.835940000

H -5.298005000 -0.501019000 -1.761909000

H -2.066868000 2.639095000 0.073154000

H -4.621438000 -2.276022000 -0.175668000

H -2.727252000 4.138323000 -1.697302000

H -3.136484000 3.242132000 -3.173233000

H -4.427304000 3.927215000 -2.183405000

H -4.683329000 -0.657567000 2.137748000

H -2.132534000 -3.895784000 2.120764000

H -3.688182000 -3.250987000 2.663874000

H -3.601996000 -4.196482000 1.166082000

H -1.902312000 -1.862834000 -0.778966000

H 0.421706000 -1.448884000 0.044828000

H 2.804193000 -3.722062000 -0.617987000

H 4.753892000 0.730316000 -0.496131000

H 1.881448000 -1.241819000 -3.462043000

H 0.450868000 -2.276879000 -3.624640000

H 0.380885000 -0.576938000 -4.131126000

H -0.389479000 -3.280348000 -0.672216000

H -1.373975000 0.048007000 -2.046237000

H 6.681874000 -1.882425000 0.773843000

H 6.987328000 -1.930194000 -0.962874000

H 6.885701000 -0.364647000 -0.126611000

H 2.320012000 4.770908000 0.182042000

H 2.462920000 4.385891000 1.901752000

H 3.786774000 3.982598000 0.798989000

H 0.653101000 -0.761818000 3.318540000

H 2.141704000 -1.100308000 2.420760000

H 2.223699000 -0.069989000 3.831719000

H -1.119546000 0.801692000 3.199813000

H -1.682721000 2.149472000 2.214030000

H 3.483523000 2.374965000 -0.984195000

H 1.880579000 2.873470000 -1.516177000

-----------------------------------------------------------------------------------

**R6**

----------------------------------------------------------------------------------

Coordinates (Angstroms)

X Y Z

-----------------------------------------------------------------------------------

C -3.312813000 -2.409300000 -1.594918000

O -4.322281000 -1.953256000 -0.807704000

C -4.183543000 -0.740785000 -0.217319000

C -3.136328000 0.071109000 -0.424313000

C -2.092736000 -0.317384000 -1.352806000

C -2.230832000 -1.651273000 -1.866852000

C -2.990309000 1.348164000 0.347544000

C -2.338873000 2.428275000 -0.508567000

C -1.057755000 1.877109000 -1.156582000

C -1.066865000 0.545449000 -1.694949000

C -3.566112000 -3.787654000 -2.084882000

O -0.106856000 2.660498000 -1.255561000

O -4.251542000 1.744344000 0.869123000

C -3.267469000 2.932576000 -1.617957000

O -1.987452000 3.505713000 0.350281000

C 1.135486000 -2.146138000 1.831013000

C 0.068582000 -1.669366000 2.830690000

C -0.566638000 -0.403745000 2.270502000

C 0.480458000 0.626600000 1.981754000

C 1.733091000 0.178996000 1.410854000

C 2.005782000 -1.165080000 1.230216000

C 0.237707000 1.936379000 2.132950000

O 1.115589000 2.905549000 1.773964000

C 2.294617000 2.538717000 1.210384000

C 2.619841000 1.241554000 1.031577000

O 1.224524000 -3.362642000 1.650597000

C 0.700343000 -1.460625000 4.208574000

O -1.564836000 0.137203000 3.121427000

O -0.949272000 -2.656046000 2.916400000

C 3.128663000 3.709811000 0.843143000

C 1.343207000 -0.160691000 -2.161771000

C 1.615394000 -1.504049000 -1.590336000

C 3.066364000 -1.556226000 -1.104358000

C 3.585957000 -0.203969000 -1.572723000

C 2.479822000 0.571584000 -2.183333000

C 3.834406000 -2.691272000 -1.786686000

O 4.732354000 0.183468000 -1.457977000

C 2.699189000 1.942307000 -2.712880000

O 0.833158000 -2.431920000 -1.582782000

C 0.005347000 0.215767000 -2.716377000

C 3.176862000 -1.692337000 0.445691000

H -5.009640000 -0.509516000 0.436059000

H -1.446213000 -2.089754000 -2.460954000

H -2.311662000 1.153633000 1.181681000

H -3.673138000 -4.474003000 -1.240623000

H -4.496947000 -3.821568000 -2.657547000

H -2.746111000 -4.125561000 -2.715430000

H -4.081825000 2.472235000 1.481240000

H -2.752331000 3.691975000 -2.208165000

H -3.582375000 2.125151000 -2.281256000

H -4.153718000 3.377191000 -1.166047000

H -1.102373000 3.776028000 0.044710000

H -1.015096000 -0.697468000 1.311161000

H -0.674630000 2.348519000 2.529275000

H 3.588241000 1.028476000 0.608976000

H 1.480455000 -0.698301000 4.189465000

H -0.071254000 -1.153003000 4.914850000

H 1.136027000 -2.400394000 4.550920000

H -2.247562000 -0.541375000 3.213808000

H -0.535652000 -3.454929000 2.540680000

H 3.328753000 4.327094000 1.722971000

H 4.073612000 3.385964000 0.411390000

H 2.598248000 4.331612000 0.117209000

H 3.369445000 -3.645940000 -1.537778000

H 3.833969000 -2.577257000 -2.873226000

H 4.870099000 -2.699597000 -1.443422000

H 3.702388000 2.283623000 -2.458795000

H 2.601692000 1.959687000 -3.802720000

H 1.953600000 2.624511000 -2.304147000

H 0.133295000 1.090170000 -3.356185000

H -0.337783000 -0.602363000 -3.351915000

H 4.116625000 -1.221371000 0.746806000

H 3.259699000 -2.753792000 0.671162000

-----------------------------------------------------------------------------------

**R7**

----------------------------------------------------------------------------------

Coordinates (Angstroms)

X Y Z

-----------------------------------------------------------------------------------

C -4.305311000 2.091786000 -0.520454000

O -5.031538000 1.025690000 -0.945344000

C -4.563696000 -0.218907000 -0.685959000

C -3.463077000 -0.467578000 0.041352000

C -2.717340000 0.636084000 0.608649000

C -3.187421000 1.933294000 0.217675000

C -2.945457000 -1.879042000 0.168427000

C -2.240363000 -2.082114000 1.507352000

C -1.296332000 -0.906492000 1.838157000

C -1.642116000 0.427318000 1.456607000

C -4.890319000 3.381563000 -0.964792000

O -0.293911000 -1.197436000 2.504958000

O -3.931670000 -2.862189000 -0.103843000

C -3.222741000 -2.236127000 2.671934000

O -1.427690000 -3.257647000 1.410487000

C 0.614162000 0.234270000 -2.105185000

C 0.199344000 -1.245073000 -2.249638000

C 0.562894000 -1.951783000 -0.943601000

C 2.033106000 -1.772922000 -0.686804000

C 2.604390000 -0.458962000 -0.890289000

C 1.862423000 0.559026000 -1.464439000

C 2.785757000 -2.767699000 -0.199473000

O 4.092608000 -2.620190000 0.138944000

C 4.651263000 -1.385764000 0.041486000

C 3.958475000 -0.328914000 -0.432464000

O -0.144991000 1.072530000 -2.600759000

C 0.874478000 -1.878547000 -3.467560000

O 0.169165000 -3.305956000 -0.927053000

O -1.207493000 -1.278620000 -2.428274000

C 6.062479000 -1.372569000 0.503353000

C 0.458240000 1.882355000 1.448080000

C 0.528905000 2.711745000 0.215492000

C 1.989613000 2.837622000 -0.221622000

C 2.702229000 2.259727000 0.995277000

C 1.708072000 1.632472000 1.900656000

C 2.375676000 4.297010000 -0.457646000

O 3.895895000 2.326734000 1.210761000

C 2.137833000 0.918683000 3.130803000

O -0.410407000 3.276208000 -0.302917000

C -0.847097000 1.545453000 2.104730000

C 2.299105000 1.999415000 -1.499989000

H -5.169916000 -0.982863000 -1.147506000

H -2.627987000 2.815804000 0.479639000

H -2.204868000 -2.004203000 -0.622623000

H -4.283413000 4.215087000 -0.616800000

H -4.947614000 3.412494000 -2.056062000

H -5.907099000 3.491377000 -0.577699000

H -4.645478000 -2.767586000 0.541183000

H -2.673064000 -2.308110000 3.611297000

H -3.905738000 -1.386042000 2.734712000

H -3.799050000 -3.153775000 2.546161000

H -0.683343000 -3.069464000 2.014288000

H 0.046947000 -1.400615000 -0.151835000

H 2.424580000 -3.767956000 -0.020053000

H 4.464060000 0.622364000 -0.469855000

H 1.962497000 -1.877750000 -3.386775000

H 0.529626000 -2.908812000 -3.563905000

H 0.589714000 -1.328144000 -4.365708000

H -0.466504000 -3.414361000 -0.194718000

H -1.429207000 -0.365572000 -2.691436000

H 6.480540000 -0.370959000 0.424029000

H 6.121527000 -1.702296000 1.544096000

H 6.665265000 -2.061243000 -0.094902000

H 3.435486000 4.368469000 -0.706363000

H 1.789332000 4.707340000 -1.280660000

H 2.191539000 4.904439000 0.431321000

H 3.109376000 0.449312000 2.968725000

H 2.261326000 1.626309000 3.957764000

H 1.409543000 0.163223000 3.417751000

H -0.650066000 1.258371000 3.137923000

H -1.450009000 2.454963000 2.130130000

H 3.372932000 2.084339000 -1.681815000

H 1.786287000 2.490889000 -2.326677000

-----------------------------------------------------------------------------------

**R8**

----------------------------------------------------------------------------------

Coordinates (Angstroms)

X Y Z

-----------------------------------------------------------------------------------

C 5.638309000 -1.793361000 0.117980000

O 6.190010000 -0.864149000 -0.704951000

C 5.441290000 0.213615000 -1.057523000

C 4.224411000 0.455872000 -0.553014000

C 3.647148000 -0.433671000 0.427939000

C 4.412116000 -1.625849000 0.656864000

C 3.355511000 1.561023000 -1.048981000

C 2.626062000 2.252039000 0.108970000

C 1.879625000 1.188871000 0.935967000

C 2.453788000 -0.122288000 1.053273000

C 6.540151000 -2.954508000 0.330114000

O 0.840020000 1.500945000 1.538232000

O 4.099294000 2.480176000 -1.826330000

C 3.575207000 3.016186000 1.027847000

O 1.738935000 3.203603000 -0.453688000

C -1.270785000 1.053081000 -0.850499000

C -1.928725000 2.304357000 -0.234993000

C -2.915828000 1.856253000 0.849316000

C -3.911485000 0.906738000 0.275346000

C -3.401643000 -0.130474000 -0.591137000

C -2.077650000 -0.135352000 -1.008046000

C -5.213850000 0.952868000 0.588330000

O -6.118074000 0.047647000 0.136923000

C -5.673117000 -0.998615000 -0.606587000

C -4.374314000 -1.121372000 -0.952388000

O -0.109296000 1.131177000 -1.268188000

C -2.609843000 3.102196000 -1.346722000

O -3.556430000 2.955510000 1.465095000

O -0.987521000 3.173496000 0.364233000

C -6.769256000 -1.934242000 -0.964769000

C 0.457531000 -1.612092000 1.126066000

C 0.514505000 -2.063171000 -0.290361000

C -0.895071000 -2.426653000 -0.756098000

C -1.644100000 -2.427546000 0.573391000

C -0.796897000 -1.775023000 1.602408000

C -0.913668000 -3.787465000 -1.445010000

O -2.734529000 -2.918851000 0.781383000

C -1.332348000 -1.460936000 2.953221000

O 1.515180000 -2.177480000 -0.966644000

C 1.662264000 -1.113721000 1.868766000

C -1.469475000 -1.320838000 -1.709731000

H 5.933552000 0.828398000 -1.794447000

H 4.040716000 -2.411607000 1.296011000

H 2.571351000 1.093329000 -1.662820000

H 7.491701000 -2.623215000 0.754524000

H 6.083078000 -3.676714000 1.003992000

H 6.757673000 -3.443294000 -0.623442000

H 3.484555000 3.198246000 -2.034864000

H 3.001006000 3.489859000 1.824442000

H 4.318912000 2.353724000 1.472511000

H 4.093384000 3.787237000 0.457167000

H 0.966278000 2.716346000 -0.800592000

H -2.323679000 1.301374000 1.594273000

H -5.664325000 1.696047000 1.227259000

H -4.094748000 -1.985798000 -1.531857000

H -1.860142000 3.412467000 -2.074948000

H -3.375934000 2.517973000 -1.856900000

H -3.075784000 3.990870000 -0.920094000

H -2.845981000 3.578624000 1.678294000

H -0.302862000 2.648393000 0.838060000

H -7.227655000 -2.342159000 -0.059819000

H -7.550063000 -1.409800000 -1.522061000

H -6.388877000 -2.754546000 -1.570457000

H -0.353003000 -3.741285000 -2.379907000

H -0.462707000 -4.557991000 -0.815617000

H -1.941150000 -4.082248000 -1.663091000

H -1.141319000 -2.284554000 3.648841000

H -0.869568000 -0.563024000 3.363664000

H -2.413115000 -1.323823000 2.906166000

H 1.337541000 -0.656820000 2.803947000

H 2.275047000 -1.977133000 2.135588000

H -2.208667000 -1.803580000 -2.349100000

H -0.655097000 -0.987409000 -2.352888000

-----------------------------------------------------------------------------------

**(7*R*,8*S*,7'*R*,8'*S*,15*S*)-3:**

**S1**

----------------------------------------------------------------------------------

Coordinates (Angstroms)

X Y Z

-----------------------------------------------------------------------------------

C -3.317354000 2.467934000 0.339824000

O -4.282195000 1.676057000 0.880063000

C -4.389935000 0.396782000 0.438173000

C -3.552571000 -0.150493000 -0.454695000

C -2.434243000 0.615347000 -0.965864000

C -2.412823000 1.984285000 -0.536377000

C -3.819122000 -1.499691000 -1.050924000

C -2.510269000 -2.244855000 -1.290219000

C -1.584807000 -1.347159000 -2.132965000

C -1.516010000 0.060683000 -1.841875000

C -3.419028000 3.875581000 0.804242000

O -0.926056000 -1.907121000 -3.016221000

O -4.725629000 -2.224095000 -0.238019000

C -1.816802000 -2.634771000 0.011257000

O -2.813332000 -3.412939000 -2.038898000

C 3.242458000 -0.774451000 0.833811000

C 2.788118000 -2.055535000 1.550272000

C 1.267746000 -2.127696000 1.486212000

C 0.678076000 -0.877355000 2.055978000

C 1.271591000 0.387354000 1.671099000

C 2.476903000 0.437353000 0.994767000

C -0.404791000 -0.904753000 2.843753000

O -1.022485000 0.213689000 3.300590000

C -0.573286000 1.422808000 2.870593000

C 0.503869000 1.536178000 2.066683000

O 4.294560000 -0.842155000 0.194525000

C 3.322439000 -2.057276000 2.984785000

O 0.736928000 -3.265534000 2.139887000

O 3.299845000 -3.176240000 0.843662000

C -1.384525000 2.537487000 3.420294000

C 0.875065000 1.035246000 -2.138724000

C 1.269332000 2.147957000 -1.239889000

C 2.772431000 2.101250000 -0.986163000

C 3.196779000 1.018888000 -1.973867000

C 1.979177000 0.342911000 -2.506406000

C 3.427977000 3.441676000 -1.319987000

O 4.322662000 0.813383000 -2.365908000

C 2.114046000 -0.835066000 -3.395444000

O 0.525298000 3.004971000 -0.800215000

C -0.533000000 0.889392000 -2.647686000

C 3.096207000 1.711602000 0.484406000

H -5.238818000 -0.111231000 0.867812000

H -1.635229000 2.648083000 -0.880151000

H -4.261269000 -1.333578000 -2.044807000

H -3.545698000 3.921402000 1.886601000

H -2.530219000 4.435614000 0.519407000

H -4.294022000 4.352532000 0.352026000

H -4.911654000 -3.052376000 -0.700229000

H -0.912144000 -3.196838000 -0.215486000

H -1.552957000 -1.760116000 0.602830000

H -2.481471000 -3.266317000 0.600650000

H -2.123992000 -3.440408000 -2.727325000

H 1.008364000 -2.154078000 0.418135000

H -0.892267000 -1.805609000 3.180202000

H 0.788524000 2.525734000 1.746160000

H 3.013573000 -2.977217000 3.481620000

H 4.412404000 -2.016248000 2.962640000

H 2.951236000 -1.207538000 3.560177000

H 1.096821000 -4.039705000 1.687008000

H 4.054723000 -2.818859000 0.342009000

H -1.254082000 2.596910000 4.504811000

H -1.084982000 3.484787000 2.977052000

H -2.444389000 2.361910000 3.227813000

H 4.509702000 3.363067000 -1.204668000

H 3.215525000 3.740666000 -2.348758000

H 3.054180000 4.219205000 -0.652317000

H 1.152857000 -1.191633000 -3.755143000

H 2.773027000 -0.594892000 -4.234513000

H 2.603719000 -1.645849000 -2.846119000

H -0.933434000 1.897648000 -2.769204000

H -0.483390000 0.438824000 -3.639900000

H 2.805071000 2.557504000 1.109320000

H 4.181625000 1.618806000 0.546233000

-----------------------------------------------------------------------------------

**S2**

----------------------------------------------------------------------------------

Coordinates (Angstroms)

X Y Z

-----------------------------------------------------------------------------------

C -1.875580000 -2.461096000 2.514298000

O -2.382201000 -3.419165000 1.688498000

C -2.889713000 -3.025436000 0.495095000

C -2.991148000 -1.747115000 0.109642000

C -2.578961000 -0.686248000 1.003802000

C -1.957379000 -1.150860000 2.215104000

C -3.371027000 -1.420368000 -1.308201000

C -4.143759000 -0.100979000 -1.377011000

C -3.481192000 1.007928000 -0.523144000

C -2.778037000 0.638086000 0.683594000

C -1.250457000 -3.049152000 3.726329000

O -3.674924000 2.184348000 -0.834166000

O -2.206677000 -1.400904000 -2.136982000

C -4.365076000 0.351900000 -2.808230000

O -5.400697000 -0.398161000 -0.737689000

C 0.897870000 -0.359121000 -0.986136000

C 1.122818000 -1.876278000 -1.105622000

C 2.173997000 -2.291909000 -0.075209000

C 3.416404000 -1.476398000 -0.230773000

C 3.262460000 -0.055532000 -0.452995000

C 2.021160000 0.496148000 -0.707597000

C 4.637846000 -2.009947000 -0.094866000

O 5.780389000 -1.276877000 -0.137005000

C 5.683514000 0.073331000 -0.257894000

C 4.489437000 0.687569000 -0.391934000

O -0.222567000 0.114059000 -1.230695000

C 1.536301000 -2.202633000 -2.543229000

O 2.474524000 -3.673203000 -0.143120000

O -0.029014000 -2.617895000 -0.735712000

C 7.014063000 0.732378000 -0.232190000

C -1.089250000 2.445073000 1.084188000

C 0.231357000 1.795527000 1.273048000

C 1.304525000 2.619054000 0.567939000

C 0.521021000 3.884250000 0.230134000

C -0.923075000 3.651003000 0.496426000

C 2.490312000 2.887785000 1.489712000

O 0.998406000 4.917116000 -0.192422000

C -1.927256000 4.686474000 0.147343000

O 0.439990000 0.811667000 1.954219000

C -2.344624000 1.789469000 1.567707000

C 1.750329000 1.970966000 -0.784370000

H -3.177311000 -3.870960000 -0.114142000

H -1.503005000 -0.452298000 2.897411000

H -4.016122000 -2.202198000 -1.712343000

H -0.426381000 -3.709271000 3.441486000

H -0.867459000 -2.265057000 4.376708000

H -1.977700000 -3.649806000 4.279391000

H -1.562932000 -0.733879000 -1.791132000

H -4.946995000 1.273413000 -2.817825000

H -3.418331000 0.542436000 -3.313000000

H -4.906394000 -0.422005000 -3.356048000

H -5.912668000 0.421634000 -0.722134000

H 1.740458000 -2.048696000 0.906389000

H 4.833000000 -3.057646000 0.071298000

H 4.486869000 1.761927000 -0.488148000

H 1.731227000 -3.271678000 -2.627726000

H 0.728505000 -1.934786000 -3.226829000

H 2.432920000 -1.657129000 -2.841446000

H 1.625499000 -4.133513000 -0.089597000

H -0.769465000 -2.395729000 -1.328852000

H 6.909501000 1.810731000 -0.336447000

H 7.527897000 0.511830000 0.707385000

H 7.639606000 0.354146000 -1.045281000

H 3.199370000 3.559723000 1.003945000

H 2.163736000 3.353477000 2.422424000

H 2.995516000 1.954905000 1.739727000

H -1.962125000 4.816035000 -0.938220000

H -2.925036000 4.421053000 0.485831000

H -1.632740000 5.651490000 0.568936000

H -2.175326000 1.439128000 2.586633000

H -3.146788000 2.526504000 1.597234000

H 2.618899000 2.530842000 -1.131553000

H 0.954653000 2.124268000 -1.513242000

-----------------------------------------------------------------------------------

**S3**

----------------------------------------------------------------------------------

Coordinates (Angstroms)

X Y Z

-----------------------------------------------------------------------------------

C -5.048435000 -1.719144000 -1.249839000

O -5.512379000 -0.602114000 -1.878358000

C -4.928067000 0.585102000 -1.585008000

C -3.973987000 0.736921000 -0.657396000

C -3.511677000 -0.404278000 0.103922000

C -4.082950000 -1.655844000 -0.312231000

C -3.276315000 2.055705000 -0.483784000

C -2.961999000 2.289793000 0.994760000

C -2.232900000 1.063809000 1.592116000

C -2.601622000 -0.252511000 1.129368000

C -5.728295000 -2.948566000 -1.731168000

O -1.441289000 1.239243000 2.522434000

O -2.099275000 2.098362000 -1.290131000

C -2.193773000 3.578109000 1.223493000

O -4.252921000 2.357385000 1.628806000

C 1.053379000 0.565276000 -0.973763000

C 1.442777000 1.994792000 -0.556540000

C 2.793357000 2.339685000 -1.190435000

C 3.815350000 1.309688000 -0.838401000

C 3.410386000 -0.075492000 -0.882644000

C 2.083020000 -0.431523000 -1.071086000

C 5.084736000 1.635221000 -0.555657000

O 6.051792000 0.715248000 -0.316100000

C 5.736767000 -0.601785000 -0.415882000

C 4.482943000 -1.009895000 -0.706280000

O -0.144103000 0.273544000 -1.122110000

C 1.492884000 2.065410000 0.973337000

O 3.245500000 3.629454000 -0.823050000

O 0.563766000 2.978644000 -1.081648000

C 6.904286000 -1.478246000 -0.145165000

C -0.608369000 -1.746148000 1.400616000

C -0.389929000 -2.410586000 0.092064000

C 1.105128000 -2.647443000 -0.110294000

C 1.672138000 -2.150094000 1.215845000

C 0.573944000 -1.578206000 2.035262000

C 1.363655000 -4.146096000 -0.295524000

O 2.828768000 -2.248157000 1.574869000

C 0.841650000 -0.988263000 3.373334000

O -1.259144000 -2.790618000 -0.664014000

C -1.986234000 -1.410883000 1.884227000

C 1.662098000 -1.854997000 -1.328464000

H -5.318212000 1.385541000 -2.197702000

H -3.739877000 -2.583540000 0.113800000

H -3.915284000 2.870520000 -0.828218000

H -5.570199000 -3.071550000 -2.806168000

H -5.343046000 -3.824964000 -1.213457000

H -6.806398000 -2.878567000 -1.562452000

H -1.574500000 1.271522000 -1.194066000

H -1.999043000 3.709625000 2.287994000

H -1.235433000 3.575480000 0.708315000

H -2.786508000 4.419974000 0.860216000

H -4.100663000 2.506944000 2.571444000

H 2.636259000 2.293819000 -2.278659000

H 5.460009000 2.644757000 -0.500618000

H 4.297058000 -2.071009000 -0.724852000

H 1.786795000 3.072660000 1.268999000

H 0.516835000 1.840635000 1.398783000

H 2.217102000 1.358955000 1.381763000

H 2.531720000 4.240912000 -1.050505000

H -0.366222000 2.696901000 -1.021834000

H 6.624080000 -2.525963000 -0.235454000

H 7.714157000 -1.261029000 -0.846736000

H 7.284868000 -1.297904000 0.863848000

H 2.436657000 -4.339877000 -0.334641000

H 0.938987000 -4.728177000 0.525701000

H 0.907430000 -4.486874000 -1.226344000

H 0.131395000 -0.193553000 3.594422000

H 0.763730000 -1.754636000 4.152151000

H 1.859175000 -0.597447000 3.413013000

H -2.607283000 -2.302386000 1.783683000

H -1.941175000 -1.156414000 2.942716000

H 0.882780000 -1.864112000 -2.093528000

H 2.505393000 -2.415218000 -1.729896000

-----------------------------------------------------------------------------------

**S4**

----------------------------------------------------------------------------------

Coordinates (Angstroms)

X Y Z

-----------------------------------------------------------------------------------

C 2.603967000 1.765849000 2.206478000

O 3.426854000 2.568266000 1.483389000

C 4.071841000 2.044358000 0.412381000

C 3.896043000 0.784104000 -0.010208000

C 2.943905000 -0.074970000 0.660299000

C 2.354751000 0.491374000 1.837915000

C 4.735621000 0.197290000 -1.105459000

C 3.904886000 -0.752448000 -1.964534000

C 3.240642000 -1.790512000 -1.042919000

C 2.665518000 -1.348931000 0.196267000

C 2.059761000 2.456668000 3.402840000

O 3.204120000 -2.957082000 -1.453921000

O 5.347378000 1.230358000 -1.857930000

C 2.837610000 -0.022981000 -2.783779000

O 4.793755000 -1.426897000 -2.842827000

C -1.421645000 1.128228000 1.272059000

C -0.979167000 2.123396000 0.189401000

C -2.225213000 2.852072000 -0.308604000

C -3.235959000 1.853705000 -0.788452000

C -3.409311000 0.628810000 -0.032644000

C -2.570649000 0.302536000 1.020180000

C -4.004441000 2.099757000 -1.858545000

O -4.984123000 1.263887000 -2.284437000

C -5.238908000 0.141384000 -1.560958000

C -4.514170000 -0.176561000 -0.468543000

O -0.734774000 1.064939000 2.296238000

C -0.239949000 1.389202000 -0.929926000

O -1.939815000 3.791146000 -1.329580000

O -0.107981000 3.077811000 0.778288000

C -6.354550000 -0.651283000 -2.136780000

C 0.343093000 -2.228932000 0.845820000

C -0.518114000 -2.048690000 2.045821000

C -1.987652000 -2.145277000 1.635483000

C -1.862692000 -2.389127000 0.135943000

C -0.429897000 -2.368252000 -0.255714000

C -2.625462000 -3.352007000 2.335031000

O -2.781709000 -2.633600000 -0.619382000

C -0.033791000 -2.559883000 -1.673568000

O -0.112187000 -1.911373000 3.180399000

C 1.834101000 -2.336186000 0.983576000

C -2.803041000 -0.861606000 1.947756000

H 4.748183000 2.751670000 -0.040971000

H 1.658248000 -0.076030000 2.435861000

H 5.508644000 -0.423922000 -0.628832000

H 1.636213000 3.425706000 3.126354000

H 1.294717000 1.848369000 3.878592000

H 2.864051000 2.650757000 4.118750000

H 5.928922000 0.800746000 -2.499461000

H 2.274082000 -0.748936000 -3.371261000

H 2.146686000 0.537362000 -2.155004000

H 3.329591000 0.672035000 -3.464327000

H 4.511720000 -2.358800000 -2.797075000

H -2.651232000 3.367810000 0.565136000

H -3.924213000 2.980618000 -2.475575000

H -4.741307000 -1.106184000 0.026524000

H 0.113500000 2.117864000 -1.659497000

H 0.617376000 0.865366000 -0.511960000

H -0.880253000 0.668917000 -1.439174000

H -1.315268000 4.425885000 -0.953224000

H 0.061310000 2.716417000 1.667200000

H -6.530554000 -1.548462000 -1.546636000

H -7.270376000 -0.054561000 -2.165019000

H -6.119014000 -0.941955000 -3.164121000

H -3.634362000 -3.512420000 1.952089000

H -2.045097000 -4.262219000 2.168592000

H -2.678028000 -3.170981000 3.409804000

H 0.115691000 -1.587265000 -2.152818000

H 0.898280000 -3.115324000 -1.765332000

H -0.831919000 -3.069301000 -2.214534000

H 2.069483000 -2.270900000 2.046857000

H 2.127478000 -3.334368000 0.647760000

H -2.539374000 -0.559606000 2.963420000

H -3.858090000 -1.134442000 1.956289000

-----------------------------------------------------------------------------------

**S5**

----------------------------------------------------------------------------------

Coordinates (Angstroms)

X Y Z

-----------------------------------------------------------------------------------

C -1.896864000 -2.407483000 2.542888000

O -2.376081000 -3.381967000 1.718263000

C -2.876155000 -3.007292000 0.516321000

C -2.987591000 -1.734764000 0.114194000

C -2.596658000 -0.656392000 1.001509000

C -1.993117000 -1.101877000 2.229866000

C -3.376100000 -1.423819000 -1.307345000

C -4.164672000 -0.108557000 -1.366559000

C -3.431049000 1.015639000 -0.595458000

C -2.787501000 0.663711000 0.655845000

C -1.281548000 -2.974700000 3.769459000

O -3.495865000 2.174975000 -0.994346000

O -2.215155000 -1.399577000 -2.142545000

C -4.484261000 0.297215000 -2.787766000

O -5.429621000 -0.312852000 -0.705476000

C 0.892716000 -0.368545000 -0.986127000

C 1.114411000 -1.885848000 -1.110227000

C 2.161458000 -2.307012000 -0.077895000

C 3.406674000 -1.494850000 -0.228956000

C 3.257627000 -0.073350000 -0.450128000

C 2.018494000 0.482799000 -0.706155000

C 4.626201000 -2.032284000 -0.090894000

O 5.771000000 -1.302708000 -0.129507000

C 5.678657000 0.047874000 -0.249693000

C 4.486766000 0.665823000 -0.386153000

O -0.226880000 0.107740000 -1.228268000

C 1.531324000 -2.208946000 -2.547563000

O 2.458162000 -3.688967000 -0.147970000

O -0.040770000 -2.625639000 -0.746590000

C 7.011125000 0.702846000 -0.220050000

C -1.085941000 2.464145000 1.061663000

C 0.223342000 1.798472000 1.263883000

C 1.312398000 2.609285000 0.568652000

C 0.549782000 3.886841000 0.230218000

C -0.899906000 3.671762000 0.483437000

C 2.497005000 2.857962000 1.497274000

O 1.046534000 4.914689000 -0.181766000

C -1.894637000 4.713075000 0.123845000

O 0.414322000 0.811631000 1.946268000

C -2.359690000 1.829522000 1.524073000

C 1.753989000 1.958780000 -0.783565000

H -3.147049000 -3.862499000 -0.087162000

H -1.559103000 -0.390522000 2.912187000

H -4.017362000 -2.213077000 -1.702477000

H -0.443118000 -3.624142000 3.502589000

H -0.920496000 -2.178943000 4.418237000

H -2.007468000 -3.582231000 4.316700000

H -1.569972000 -0.732773000 -1.798994000

H -5.042826000 1.231343000 -2.785442000

H -3.571628000 0.434970000 -3.365105000

H -5.086611000 -0.480667000 -3.261799000

H -5.265836000 -0.422956000 0.240941000

H 1.726197000 -2.064461000 0.903187000

H 4.817836000 -3.080789000 0.074281000

H 4.487739000 1.740205000 -0.481725000

H 1.724315000 -3.278161000 -2.634474000

H 0.725990000 -1.937576000 -3.232673000

H 2.429765000 -1.664429000 -2.841923000

H 1.607748000 -4.147095000 -0.098266000

H -0.778078000 -2.398057000 -1.341704000

H 6.910096000 1.781571000 -0.323891000

H 7.521755000 0.480165000 0.720772000

H 7.637691000 0.323236000 -1.031697000

H 3.219406000 3.519476000 1.017028000

H 2.172193000 3.327239000 2.428805000

H 2.986134000 1.916998000 1.748773000

H -2.235462000 4.557898000 -0.903258000

H -2.774662000 4.678530000 0.765086000

H -1.436135000 5.701716000 0.180079000

H -2.223998000 1.499129000 2.554939000

H -3.153068000 2.576912000 1.514013000

H 2.624946000 2.514556000 -1.131433000

H 0.958218000 2.115637000 -1.511661000

-----------------------------------------------------------------------------------

**S6**

----------------------------------------------------------------------------------

Coordinates (Angstroms)

X Y Z

-----------------------------------------------------------------------------------

C -5.039230000 -1.705557000 -1.274183000

O -5.494029000 -0.584899000 -1.905335000

C -4.911557000 0.599872000 -1.601891000

C -3.964746000 0.747754000 -0.665512000

C -3.513653000 -0.397921000 0.100560000

C -4.085853000 -1.647142000 -0.324372000

C -3.268704000 2.067155000 -0.473538000

C -2.974357000 2.284470000 1.016876000

C -2.219408000 1.064017000 1.591676000

C -2.611809000 -0.252435000 1.135055000

C -5.714977000 -2.931772000 -1.768365000

O -1.388314000 1.227555000 2.484164000

O -2.086042000 2.115719000 -1.272514000

C -2.236714000 3.580977000 1.270783000

O -4.225889000 2.391722000 1.721440000

C 1.056519000 0.574477000 -0.955224000

C 1.454699000 2.002561000 -0.540458000

C 2.801009000 2.341302000 -1.186906000

C 3.821807000 1.308228000 -0.840442000

C 3.411285000 -0.075296000 -0.879876000

C 2.081256000 -0.425985000 -1.061230000

C 5.094449000 1.629262000 -0.567229000

O 6.059315000 0.705760000 -0.332578000

C 5.738228000 -0.610092000 -0.426532000

C 4.480760000 -1.013744000 -0.707793000

O -0.143237000 0.286248000 -1.095603000

C 1.519557000 2.073888000 0.988688000

O 3.260935000 3.630142000 -0.826676000

O 0.575773000 2.990516000 -1.057865000

C 6.903799000 -1.490787000 -0.161246000

C -0.620927000 -1.749547000 1.403994000

C -0.401729000 -2.403474000 0.090615000

C 1.092767000 -2.643484000 -0.110404000

C 1.658973000 -2.156653000 1.219716000

C 0.559965000 -1.591324000 2.043600000

C 1.346774000 -4.142145000 -0.303089000

O 2.815450000 -2.256646000 1.578097000

C 0.828049000 -1.016895000 3.387966000

O -1.270772000 -2.774830000 -0.670229000

C -1.999751000 -1.414667000 1.886741000

C 1.654272000 -1.846838000 -1.323548000

H -5.292425000 1.403112000 -2.216910000

H -3.749390000 -2.576786000 0.102586000

H -3.905439000 2.884923000 -0.814555000

H -5.541583000 -3.051595000 -2.841334000

H -5.339016000 -3.810522000 -1.247905000

H -6.795248000 -2.859595000 -1.615229000

H -1.557158000 1.290906000 -1.175918000

H -2.038344000 3.686354000 2.335649000

H -1.288889000 3.607515000 0.738497000

H -2.852354000 4.418193000 0.935561000

H -4.670552000 1.534010000 1.693227000

H 2.634749000 2.293168000 -2.273731000

H 5.474225000 2.637355000 -0.516859000

H 4.290183000 -2.074114000 -0.722082000

H 1.825860000 3.078626000 1.280325000

H 0.545208000 1.858778000 1.422779000

H 2.240791000 1.360981000 1.391266000

H 2.545127000 4.242887000 -1.044239000

H -0.355319000 2.715829000 -0.985715000

H 7.709084000 -1.280253000 -0.870106000

H 7.293181000 -1.307958000 0.843939000

H 6.618117000 -2.537565000 -0.245037000

H 2.419240000 -4.339040000 -0.341131000

H 0.918760000 -4.727226000 0.514272000

H 0.891325000 -4.476542000 -1.236599000

H 0.759274000 -1.794313000 4.156627000

H 1.842963000 -0.619324000 3.428818000

H 0.114213000 -0.229623000 3.622261000

H -2.621743000 -2.305037000 1.781834000

H -1.955869000 -1.164368000 2.946285000

H 0.875549000 -1.849542000 -2.089363000

H 2.495465000 -2.408748000 -1.727026000

-----------------------------------------------------------------------------------

**S7**

----------------------------------------------------------------------------------

Coordinates (Angstroms)

X Y Z

-----------------------------------------------------------------------------------

C -3.062124000 2.021036000 -1.922328000

O -4.061026000 1.960774000 -1.003468000

C -4.269707000 0.792081000 -0.348662000

C -3.499375000 -0.292677000 -0.512075000

C -2.358926000 -0.244571000 -1.402863000

C -2.247621000 0.970650000 -2.153526000

C -3.824472000 -1.595539000 0.151988000

C -2.541055000 -2.267127000 0.631242000

C -1.582052000 -2.395956000 -0.565794000

C -1.472810000 -1.305795000 -1.494929000

C -2.969191000 3.357480000 -2.561095000

O -0.922089000 -3.439237000 -0.636441000

O -4.762362000 -1.393465000 1.194007000

C -1.857791000 -1.496812000 1.760115000

O -2.877993000 -3.570725000 1.084251000

C 1.701796000 2.685472000 0.467269000

C 0.454487000 2.885143000 1.343831000

C 0.705078000 2.256046000 2.711444000

C 1.131266000 0.828259000 2.554022000

C 2.049262000 0.499376000 1.480471000

C 2.367683000 1.402196000 0.487292000

C 0.729451000 -0.122272000 3.407330000

O 1.159246000 -1.410972000 3.351575000

C 2.122108000 -1.732958000 2.447490000

C 2.589366000 -0.830067000 1.561995000

O 2.039348000 3.633780000 -0.243114000

C -0.755378000 2.287925000 0.626217000

O -0.422787000 2.337020000 3.566293000

O 0.248504000 4.276780000 1.539562000

C 2.536304000 -3.155050000 2.549061000

C 0.988033000 -1.096758000 -2.154278000

C 1.504866000 0.301264000 -2.140116000

C 2.994639000 0.286705000 -1.796633000

C 3.232476000 -1.199145000 -1.567272000

C 1.987002000 -1.955329000 -1.846400000

C 3.801140000 0.726470000 -3.031440000

O 4.287691000 -1.699678000 -1.231262000

C 1.995476000 -3.438621000 -1.798483000

O 0.865599000 1.287820000 -2.440959000

C -0.415464000 -1.419924000 -2.576080000

C 3.405854000 1.159097000 -0.576078000

H -5.126450000 0.838843000 0.304733000

H -1.427616000 1.112219000 -2.838379000

H -4.252233000 -2.261540000 -0.612386000

H -2.819408000 4.127159000 -1.799225000

H -2.137717000 3.386919000 -3.262518000

H -3.896146000 3.594306000 -3.090588000

H -4.961640000 -2.265152000 1.560782000

H -0.965295000 -2.038841000 2.069304000

H -1.573898000 -0.488966000 1.461534000

H -2.536820000 -1.426709000 2.610170000

H -2.183295000 -4.140142000 0.706048000

H 1.547718000 2.809179000 3.152222000

H 0.031712000 0.037239000 4.213600000

H 3.368402000 -1.150083000 0.890454000

H -1.648606000 2.434376000 1.233473000

H -0.883113000 2.798535000 -0.326171000

H -0.631226000 1.223961000 0.432900000

H -0.624551000 3.276607000 3.669500000

H 0.787714000 4.694619000 0.843044000

H 2.843385000 -3.392729000 3.570542000

H 1.696515000 -3.808365000 2.295086000

H 3.360591000 -3.360652000 1.868731000

H 4.868699000 0.675159000 -2.811415000

H 3.596185000 0.087200000 -3.893345000

H 3.539255000 1.753365000 -3.288831000

H 2.332038000 -3.774499000 -0.813727000

H 1.013048000 -3.860404000 -1.985402000

H 2.719030000 -3.829027000 -2.520491000

H -0.673829000 -0.767611000 -3.411063000

H -0.435507000 -2.445418000 -2.947722000

H 3.692090000 2.137395000 -0.958550000

H 4.305991000 0.702687000 -0.156144000

-----------------------------------------------------------------------------------

**References**

1. Wang, H.-H.; Li, G.; Qiao, Y.-N.; Sun, Y.; Peng, X.-P.; Lou, H.-X. *Org. Lett.* **2019**, *21*, 3319-3322.
