# Supplementary material for: A Novel Azaphilone Muyophilone A From the Endophytic Fungus Muyocopron laterale 0307-2
Source: Front Chem. 2021 Aug 24;9:734822. doi: 10.3389/fchem.2021.734822 (PMC8417894; doi:10.3389/fchem.2021.734822)

checkCIF/PLATON report

Structure factors have been supplied for datablock(s) cu_20200610_0ma_a

THIS REPORT IS FOR GUIDANCE ONLY. IF USED AS PART OF A REVIEW PROCEDURE FOR PUBLICATION, IT SHOULD NOT REPLACE THE EXPERTISE OF AN EXPERIENCED CRYSTALLOGRAPHIC REFEREE.

No syntax errors found. CIF dictionary Interpreting this report

Datablock: cu_20200610_0ma_a

```
Bond precision:  C-C = 0.0048 A         Wavelength=1.54178

Cell:             a=8.4390(4)      b=19.938(1)      c=6.4295(4)
                  alpha=90         beta=90          gamma=90
Temperature:      296 K

                Calculated                 Reported
Volume          1081.81(10)                1081.81(10)
Space group     P 21 21 2                  P 21 21 2
Hall group      P 2 2ab                    P 2 2ab
Moiety formula  C12 H12 O5                 C12 H12 O5
Sum formula     C12 H12 O5                 C12 H12 O5
Mr              236.22                     236.22
Dx,g cm-3       1.450                      1.450
Z               4                          4
Mu (mm-1)       0.964                      0.964
F000            496.0                      496.0
F000'           497.80
h,k,lmax        10,24,7                    10,24,7
Nref            2001[ 1192]                1997
Tmin,Tmax                                  0.503,0.753
Tmin'

Correction method= # Reported T Limits: Tmin=0.503 Tmax=0.753
AbsCorr = NONE

Data completeness= 1.68/1.00      Theta(max)= 68.367

R(reflections)= 0.0564( 1831)     wR2(reflections)= 0.1519( 1997)

S = 1.070                  Npar= 156
```

The following ALERTS were generated. Each ALERT has the format
test-name_ALERT_alert-type_alert-level.
Click on the hyperlinks for more details of the test.

Alert level A

```
EXPT005_ALERT_1_A  _exptl_crystal_description is missing
            Crystal habit description.
            The following tests will not be performed.
            CRYSR_01
PLAT414_ALERT_2_A Short Intra D-H..H-X       H003     ..H00B            1.66 Ang.
                                                      x,y,z  =      1_555 Check
PLAT699_ALERT_1_A Missing _exptl_crystal_description Value .......     Please Do !
```

Alert level B

```
PLAT035_ALERT_1_B _chemical_absolute_configuration Info  Not Given     Please Do !
PLAT420_ALERT_2_B D-H Without Acceptor       O003     --H003     .     Please Check
PLAT430_ALERT_2_B Short Inter D...A Contact  O005     ..O005     .       2.77 Ang.
                                                  1-x,1-y,z  =      2_665 Check
```

Alert level C

```
PLAT053_ALERT_1_C Minimum Crystal Dimension Missing (or Error) ...     Please Check
PLAT054_ALERT_1_C Medium  Crystal Dimension Missing (or Error) ...     Please Check
PLAT055_ALERT_1_C Maximum Crystal Dimension Missing (or Error) ...     Please Check
PLAT340_ALERT_3_C Low Bond Precision on  C-C Bonds ...............    0.00483 Ang.
PLAT415_ALERT_2_C Short Inter D-H..H-X       H003     ..H00F     .       2.11 Ang.
                                                   -1+x,y,z  =      1_455 Check
PLAT976_ALERT_2_C Check Calcd Resid. Dens.  1.06A   From O003          -0.43 eA-3
PLAT976_ALERT_2_C Check Calcd Resid. Dens.  0.89A   From O004          -0.42 eA-3
PLAT977_ALERT_2_C Check Negative Difference Density on H003            -0.36 eA-3
PLAT977_ALERT_2_C Check Negative Difference Density on H004            -0.42 eA-3
```

Alert level G

```
PLAT007_ALERT_5_G Number of Unrefined Donor-H Atoms ..............          2 Report
PLAT720_ALERT_4_G Number of Unusual/Non-Standard Labels ..........         29 Note
PLAT791_ALERT_4_G Model has Chirality at C00C        (Sohnke SpGr)          S Verify
PLAT791_ALERT_4_G Model has Chirality at C00E        (Sohnke SpGr)          R Verify
PLAT883_ALERT_1_G No Info/Value for _atom_sites_solution_primary .     Please Do !
PLAT912_ALERT_4_G Missing # of FCF Reflections Above STh/L=  0.600          1 Note
PLAT978_ALERT_2_G Number C-C Bonds with Positive Residual Density.          0 Info
```

3 **ALERT level A** = Most likely a serious problem - resolve or explain
3 **ALERT level B** = A potentially serious problem, consider carefully
9 **ALERT level C** = Check. Ensure it is not caused by an omission or oversight
7 **ALERT level G** = General information/check it is not something unexpected

7 ALERT type 1 CIF construction/syntax error, inconsistent or missing data
9 ALERT type 2 Indicator that the structure model may be wrong or deficient
1 ALERT type 3 Indicator that the structure quality may be low
4 ALERT type 4 Improvement, methodology, query or suggestion
1 ALERT type 5 Informative message, check

It is advisable to attempt to resolve as many as possible of the alerts in all categories. Often the minor alerts point to easily fixed oversights, errors and omissions in your CIF or refinement strategy, so attention to these fine details can be worthwhile. In order to resolve some of the more serious problems it may be necessary to carry out additional measurements or structure refinements. However, the purpose of your study may justify the reported deviations and the more serious of these should normally be commented upon in the discussion or experimental section of a paper or in the "special_details" fields of the CIF. checkCIF was carefully designed to identify outliers and unusual parameters, but every test has its limitations and alerts that are not important in a particular case may appear. Conversely, the absence of alerts does not guarantee there are no aspects of the results needing attention. It is up to the individual to critically assess their own results and, if necessary, seek expert advice.

Publication of your CIF in IUCr journals

A basic structural check has been run on your CIF. These basic checks will be run on all CIFs submitted for publication in IUCr journals (*Acta Crystallographica*, *Journal of Applied Crystallography*, *Journal of Synchrotron Radiation*); however, if you intend to submit to *Acta Crystallographica Section C* or *E* or *IUCrData*, you should make sure that full publication checks are run on the final version of your CIF prior to submission.

Publication of your CIF in other journals

Please refer to the *Notes for Authors* of the relevant journal for any special instructions relating to CIF submission.

Validation response form

Please find below a validation response form (VRF) that can be filled in and pasted into your CIF.

```
# start Validation Reply Form
_vrf_EXPT005_cu_20200610_0ma_a
;
PROBLEM: _exptl_crystal_description is missing
RESPONSE: ...
;
_vrf_PLAT414_cu_20200610_0ma_a
;
PROBLEM: Short Intra D-H..H-X       H003     ..H00B             1.66 Ang.
RESPONSE: ...
;
_vrf_PLAT699_cu_20200610_0ma_a
;
PROBLEM: Missing _exptl_crystal_description Value .......     Please Do !
RESPONSE: ...
;
_vrf_PLAT035_cu_20200610_0ma_a
;
PROBLEM: _chemical_absolute_configuration Info  Not Given     Please Do !
RESPONSE: ...
;
_vrf_PLAT420_cu_20200610_0ma_a
;
PROBLEM: D-H Without Acceptor       O003     --H003     .     Please Check
RESPONSE: ...
;
_vrf_PLAT430_cu_20200610_0ma_a
```

```
;
PROBLEM: Short Inter D...A Contact  O005     ..O005     .       2.77 Ang.
RESPONSE: ...
;
_vrf_PLAT053_cu_20200610_0ma_a
;
PROBLEM: Minimum Crystal Dimension Missing (or Error) ...     Please Check
RESPONSE: ...
;
_vrf_PLAT054_cu_20200610_0ma_a
;
PROBLEM: Medium  Crystal Dimension Missing (or Error) ...     Please Check
RESPONSE: ...
;
_vrf_PLAT055_cu_20200610_0ma_a
;
PROBLEM: Maximum Crystal Dimension Missing (or Error) ...     Please Check
RESPONSE: ...
;
_vrf_PLAT340_cu_20200610_0ma_a
;
PROBLEM: Low Bond Precision on  C-C Bonds ...............    0.00483 Ang.
RESPONSE: ...
;
_vrf_PLAT415_cu_20200610_0ma_a
;
PROBLEM: Short Inter D-H..H-X       H003     ..H00F     .       2.11 Ang.
RESPONSE: ...
;
_vrf_PLAT976_cu_20200610_0ma_a
;
PROBLEM: Check Calcd Resid. Dens.  1.06A   From O003           -0.43 eA-3
RESPONSE: ...
;
_vrf_PLAT977_cu_20200610_0ma_a
;
PROBLEM: Check Negative Difference Density on H003             -0.36 eA-3
RESPONSE: ...
;
# end Validation Reply Form
```

PLATON version of 05/12/2020; check.def file version of 05/12/2020

Datablock cu_20200610_0ma_a - ellipsoid plot

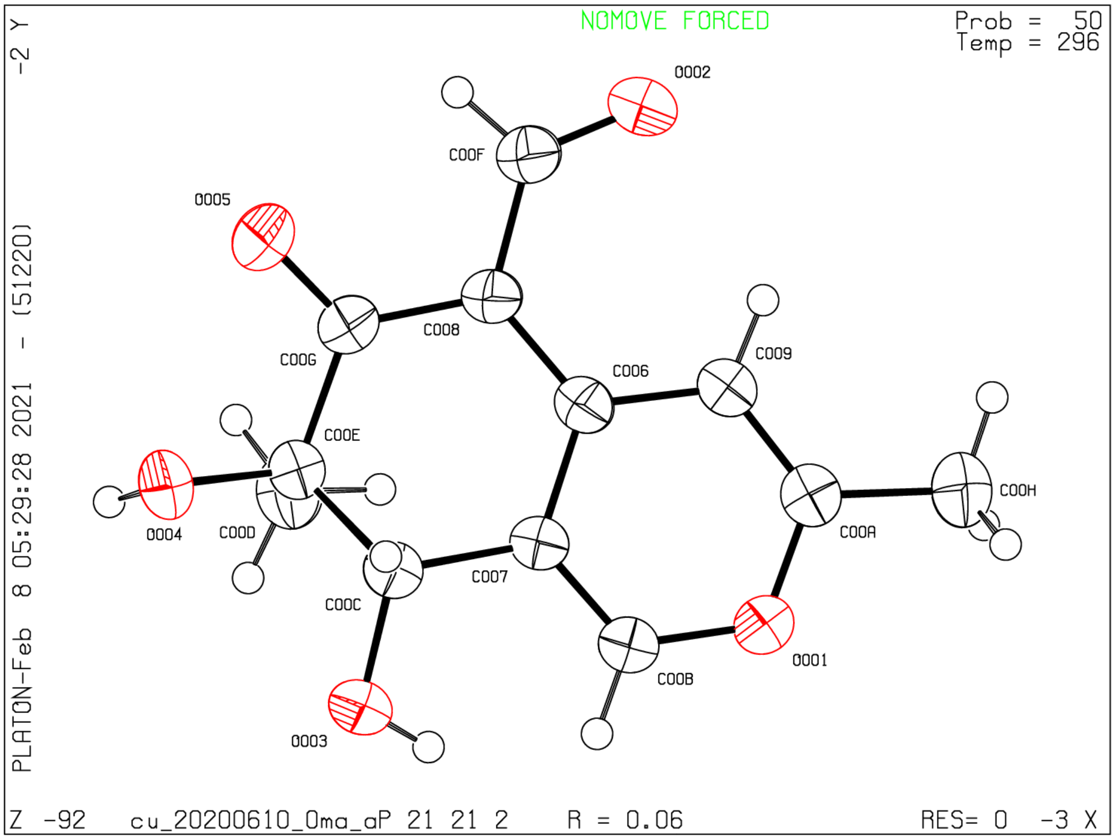

Supplement: Supplementary file 2 [file DataSheet1.PDF]
